# Supplementary material for: Quantifying cell transitions in C. elegans with data-fitted landscape models
Source: PLoS Comput Biol. 2021 Jun 1;17(6):e1009034. doi: 10.1371/journal.pcbi.1009034 (PMC8195438; doi:10.1371/journal.pcbi.1009034)
Supplement: S1 Appendix — In this note we introduce concepts from Dynamical Systems and Catastrophe Theory, give further details about the mathematical underpinnings of the methodology to create the landscape model as well as about the implementation of the ABC SMC algorithm and fitting results discussed in the main paper. (PDF) [file pcbi.1009034.s001.pdf]

Supporting Information
Quantifying cell transitions in *C. elegans* with data-fitted landscape models

Elena Camacho-Aguilar[1,2*], Aryeh Warmflash[2,3], and David A. Rand[1,4*]

[1]Mathematics Institute, University of Warwick, Coventry, United Kingdom, [2]Department of Biosciences, Rice University, Houston, Texas, United States of America, [3]Department of Bioengineering, Rice University, Houston, Texas, United States of America, [4]Zeeman Institute for Systems Biology & Infectious Disease Epidemiology Research, University of Warwick, Coventry, United Kingdom

*ec47@rice.edu (ECA), d.a.rand@warwick.ac.uk (DAR)

Contents

1 Concepts from Dynamical Systems and Catastrophe Theory 2
1.1 Fold catastrophe . . . 2
1.2 Cusp catastrophe . . . 3

2 Details of the mathematical model: Binary flip with cusp landscape model 5
2.1 Stability . . . 5
2.2 Bifurcation set . . . 7
2.2.1 Non-genericity of flip endpoint . . . 10
2.3 Dependence upon the morphogens . . . 10
2.3.1 Type I transformations . . . 13
2.3.2 Type II transformations . . . 14
2.4 Model implementation and numerical simulations . . . 15
2.4.1 Initial condition . . . 17
2.4.2 Simulation procedure . . . 18
2.4.3 Fate assignment . . . 19
2.4.4 Simulation of mutants . . . 20

3 Parameter estimation 22
3.1 Constraints on the parameters . . . 24
3.1.1 Constraint imposed by the sigmoidal function $\chi(y)$ . . . 24
3.1.2 Constraint imposed by mutant (5) Notch null, 2xWT EGF . . . 26
3.1.3 Constraint imposed by mutant (6) Notch null, WT EGF . . . 26
3.2 Ranges and priors . . . 26
3.3 Distance . . . 27
3.4 Sequence of thresholds and number of particles . . . 28
3.5 Perturbation kernel . . . 28

4 Fitting results 29
4.1 Software run-time . . . 29
4.2 Approximated posteriors . . . 29
4.3 Exploring the single-cell fate map . . . 29

1 Concepts from Dynamical Systems and Catastrophe Theory

Here we will provide some basic concepts in the fields of catastrophe and bifurcation theory for readers unfamiliar with them. Most of the concepts summarised in this section are taken from the book [1], where we refer the reader if interested in more details.

Let V be a family of functions which, in our settings, will represent the familly of potential functions defining a landscape model:

$$V : S \times C \to \mathbb{R}$$

where S is a manifold in $\mathbb{R}^n$ and C is another manifold in $\mathbb{R}^r$. Let us call $\mathbb{R}^n$ the **state space** and $\mathbb{R}^r$ the **control or parameter space**. For a fixed value $\boldsymbol{c}$ in C, we will denote by $V_{\boldsymbol{c}} : S \to \mathbb{R}$ the potential function $V_{\boldsymbol{c}}(\boldsymbol{x}) = V(\boldsymbol{x}, \boldsymbol{c})$ for $\boldsymbol{x} \in S$.

For a fixed value $\boldsymbol{c}$ for the **control variables** or **parameters**, the function $V_{\boldsymbol{c}}(\boldsymbol{x})$ will have certain critical points. Those will be given by the points $\boldsymbol{x}^*$ such that $D_X V_{\boldsymbol{c}}(\boldsymbol{x}^*) = 0$, i.e. the gradient of the potential at that particular point is zero. The critical point is **degenerate** if the rank of the Hessian matrix of $V_{\boldsymbol{c}}$ evaluated at $\boldsymbol{x}^*$ is not maximum, i.e. $\det(D^2_X V_{\boldsymbol{c}}(\boldsymbol{x}^*)) = 0$. If the critical point is not degenerate, we say that it is **non-degenerate**. We are interested in knowing how the number of critical points and their stability change depending on the value $\boldsymbol{c}$, the parameters.

Let us define the **catastrophe manifold** $\mathcal{M}$ as the subset of $\mathbb{R}^n \times \mathbb{R}^r$ defined by:

$$\mathcal{M} = \{(\boldsymbol{x}, c) \in \mathbb{R}^n \times \mathbb{R}^r : D_X V_{\boldsymbol{c}}(\boldsymbol{x}) = 0\} \tag{1}$$

where D_X is the gradient in the X variables. In other words, $\mathcal{M}$ is the set of points $(\boldsymbol{x}, \boldsymbol{c})$ such that $\boldsymbol{x}$ is a critical point of the function $V_{\boldsymbol{c}}$, i.e. it contains all the critical points of the family of functions V.

The **catastrophe map** χ is the restriction to $\mathcal{M}$ of the natural projection

$$\begin{aligned} \pi : \mathbb{R}^n \times \mathbb{R}^r &\to \mathbb{R}^r \\ \pi(\boldsymbol{x}, C) &= C \end{aligned} \tag{2}$$

The **singularity set** $\mathcal{S}$ is the set of singular points in $\mathcal{M}$ at which χ is singular, that is, where the rank of the derivative $D\chi$ is less than r. Actually, it is not hard to show that $\mathcal{S}$ is the set of points $(\boldsymbol{x}, \boldsymbol{c}) \in \mathcal{M}$ at which $V_{\boldsymbol{c}}(\boldsymbol{x})$ has a degenerate critical point. The image $\chi(\mathcal{S})$ in C is called the **bifurcation set**. It follows that $\mathcal{B}$ is the set on which the number and nature of the critical points change.

Summing up, the catastrophe manifold gives a description of the critical points for the family of functions $V_{\boldsymbol{c}}(\boldsymbol{x})$, and thus it describes how they change as the parameters change. The bifurcation set describes the set of parameters for which important changes in the critical points take place.

In what follows, we will introduce these concepts in the context of two particular families of functions, the fold and the cusp, which we will use to build the binary flip with cusp landscape model.

1.1 Fold catastrophe

The fold is the most basic catastrophe and it has the form:

$$V(x, c) = V_c(x) = \frac{x^3}{3} + cx. \tag{3}$$

It is a cubic equation with graph always crossing the point $x = 0$. As we will see, depending on the value of the control parameter c, the potential function V_c will have two, one or no critical points (Fig. SI1A1-4).

The catastrophe manifold $\mathcal{M}$ is given by the critical points of the potential function for each value of c:

$$0 = \frac{dV_c(x)}{dx} = -f_{\text{fold}} = x^2 + c = 0. \tag{4}$$

Therefore we can use the x-coordinate as a chart for $\mathcal{M}$ and write it as

$$\mathcal{M} = \{(x, c) \in \mathbb{R} \times \mathbb{R} : c = -x^2\} = \{(x, -x^2) \in \mathbb{R} \times \mathbb{R}_{\leq 0}\}. \tag{5}$$

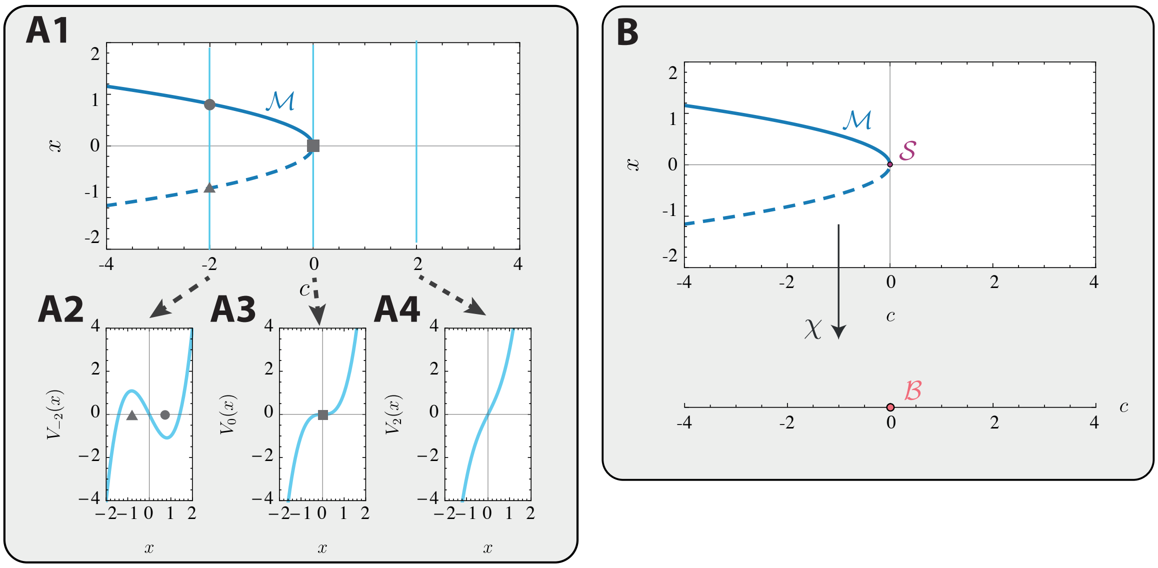

Figure SI1: (A1) Bifurcation diagram or catastrophe manifold of the fold catastrophe. Catastrophe manifold $\mathcal{M}$. (A2-4) Plots of the fold potential for the corresponding values of the control parameter c. Continuous line in the bifurcation diagram and gray circle represent stable points, dashed line and gray triangle represent unstable points, gray square represents degenerate point (A2) In this case $c = -2$, for which the potential has two critical points, a minimum and a maximum. (A3) In this case $c = 0$ and corresponds to a bifurcation point. The potential function only contains a degenerate critical point at $x = 0$. (A4) In this case $c = 2$ and the potential function does not contain any critical point. (B) Catastrophe manifold $\mathcal{M}$, catastrophe map χ, singularity set $\mathcal{S}$ and bifurcation set $\mathcal{B}$ of the fold catastrophe given in Eq. 5. Again, continuous line represents stable points, dashed line represents unstable points.

This means that for each value of c, the potential function has two critical points ($c < 0$), one critical point ($c = 0$) or no critical points ($c > 0$) (Fig. SI1A1).

If we look at the stability of such critical points, $D^2V_c(x) = \frac{d^2V_c(x)}{dx^2} = 2x$, which evaluated at the points $(x, -x^2)$ of the catastrophe manifold, is equal to $2x$, which sign depends on the sign of x. If $x < 0$, the critical point is unstable. If $x > 0$, the critical point is stable. And if $x = 0$ the critical point is degenerate (Fig. SI1A1-4). In fact, the singularity set is the set of points $(x, c) \in \mathcal{M}$ such that

$$0 = D^2V(x, c) = D^2V(x, -x^2) = \frac{d^2V}{dx^2}(x, -x^2) = -2x \iff x = c = 0. \tag{6}$$

Hence, the singularity set $\mathcal{S}$ of the fold catastrophe is the point $(0, 0)$ and the bifurcation set $\mathcal{B}$ is the point $c = 0$ in the control space (Fig. SI1B).

The catastrophe manifold helps us to identify regions in the control space for which the potential function will have the same critical points. For example, for the fold, if $c < 0$, the potential will contain two critical points ($x_1^* = -\sqrt{-c}/3$ and $x_2^* = \sqrt{-c}/3$); if $c = 0$, the potential has just one degenerate critical point ($x^* = 0$); and if $c > 0$ the potential function $V_c(x)$ contains no critical points (see Fig. SI1).

1.2 Cusp catastrophe

This catastrophe has the form

$$V_{a,b}(x) = x^4 + ax^2 + bx, \tag{7}$$

which is a polynomial of order 4 in one variable. In this case, the control space is 2-dimensional on a, b, and depending on their values, this potential function will contain one, two or three critical points (see Fig. SI2A1-4).

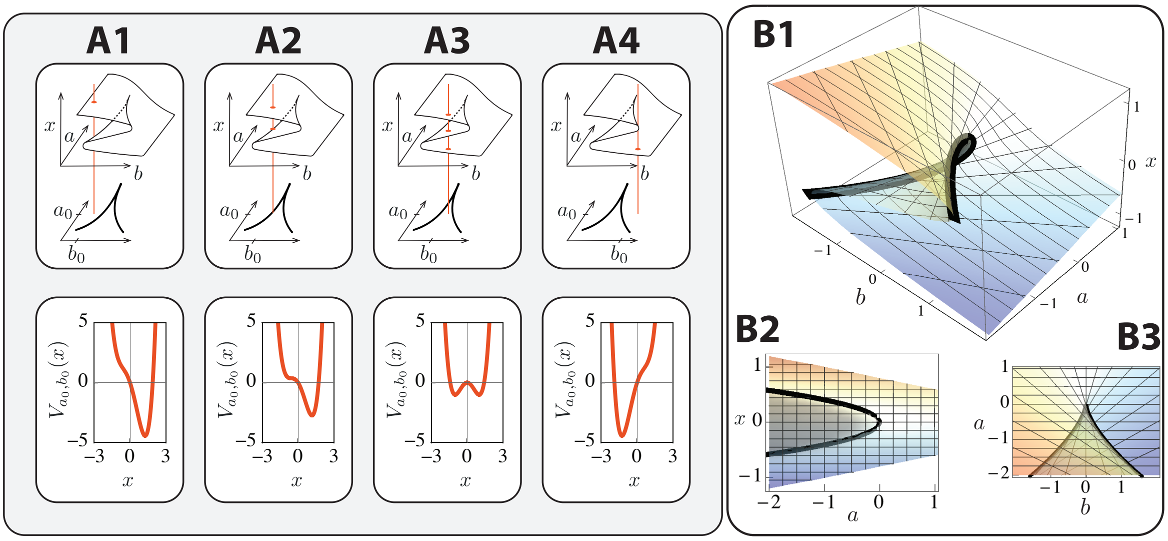

Figure SI2: (A1-4) Four different potential functions from the cusp family for control parameters in different regions of the control space. Each row of plots shows, on the top figure, the catastrophe manifold intersected with the line (in orange) $L_{a_0,b_0} = \{(x, a_0, b_0) \in \mathbb{R} \times \mathbb{R}^2 : x \in \mathbb{R}\}$ for a fixed value $a_0 < 0$ and different values of b_0, displaying the critical points (orange ellipses); and, on the bottom figure, the potential function corresponding to such values of a and b with the predicted critical points. (A1) The control parameters are such that $8a_0^3 + 27b_0^2 > 0$ and $b_0 < 0$, therefore the potential has only one critical point with $x > 0$. (A2) In this case $(a_0, b_0) \in \mathcal{B}$ and $b_0 > 0$, so the potential has two critical points, one of them is degenerate. (A3) The control parameters are such that $8a_0^3 + 27b_0^2 < 0$ so the potential function has three critical points. (A4) Similar to (A1), The control parameters are such that $8a_0^3 + 27b_0^2 > 0$ but now $b_0 > 0$, therefore the potential has one, now negative, critical point. (B1) Catastrophe manifold $\mathcal{M}$ of the cusp catastrophe. Singularity set represented as a thick black line. (B2) Different view of the catastrophe manifold. Projection onto the (x, a) space. (B3) Projection of the singularity set onto the control space. The thick black line represents the bifurcation set of the cusp in the control space.

The catastrophe manifold $\mathcal{M}$ is given by the points in $\mathbb{R} \times \mathbb{R}^2$ such that

$$0 = \frac{dV_{a,b}(x)}{dx} = 4x^3 + 2ax + b = -f_{\text{cusp}} = 0. \tag{8}$$

Hence, we can use (x, a) as a chart on $\mathcal{M}$ and write b in terms of x and a as $b = -4x^3 - 2ax$. This allows us to write the catastrophe manifold as the set of points

$$\mathcal{M} = \{(x, a, b) \in \mathbb{R} \times \mathbb{R}^2 : b = -4x^3 - 2ax\} = \{(x, a, -4x^3 - 2ax) \in \mathbb{R} \times \mathbb{R}^2\}. \tag{9}$$

Fig. SI2B1 contains a plot of this two dimensional manifold in $\mathbb{R}^3$.

The singularity set $\mathcal{S}$ of the cusp catastrophe is given by the points in the catastrophe manifold such that

$$D^2V(x, a, -4x^3 - 2ax) = \frac{d^2V}{dx^2}(x, a, -4x^3 - 2ax) = 12x^2 + 2a = 0.$$

Therefore, $a = -6x^2$; and since $b = -4x^3 - 2ax$ in $\mathcal{M}$, then

$$\mathcal{S} = \{(x, a, b) \in \mathcal{M} : a = -6x^2\} = \{(x, -6x^2, 8x^3) \in \mathbb{R} \times \mathbb{R}^2, x \in \mathbb{R}\}, \tag{10}$$

which is a curve in $\mathbb{R}^3$ (see Fig. SI2B1).

Finally, the bifurcation set $\mathcal{B}$ is given by the image of this set by the catastrophe map, which is the projection of this curve on the control space:

$$\mathcal{B} = \{(-6t^2, 8t^3) \in \mathbb{R}^2, t \in \mathbb{R}\} = \{(a, b) \in \mathbb{R}^2 : \Delta(a, b) = 8a^3 + 27b^2 = 0\}. \tag{11}$$

As we see in Fig. SI2B3, the bifurcation set defines three regions in the control space. For values of the control parameters (a_0, b_0) in the region such that $\Delta(a_0, b_0) = 8a_0^3 + 27b^2 < 0$, the potential function contains three critical points (two stable and one unstable). If (a_0, b_0) is in the bifurcation set, the potential function contains two critical points (one stable and one degenerate). And if the control parameters are in the region such that $\Delta(a_0, b_0) > 0$, then the potential function contains just one stable critical point (see Fig. SI2). Since the critical points are given by the zeros of a third-degree polynomial, the critical points in each case can be easily computed with Cardano's formula.

2 Details of the mathematical model: Binary flip with cusp landscape model

2.1 Stability

It follows from CT that these two bifurcations are universal for gradient-like systems in the following sense. Suppose a gradient-like system $\dot{x} = f(x, \theta)$ with state $x = (x_1, \ldots, x_n)$ and parameters $\theta = (\theta_1, \ldots, \theta_d)$ has a bifurcation at $x = x^*$, $\theta = \theta^*$ that is generic for 1-parameter (resp. 2-parameter) systems, then for (x, θ) near (x^*, θ^*), f is induced from the normal form $g = f_{\text{fold}}$ (resp. $g = f_{\text{cusp}}$) for the saddle-node (resp. cusp) in that there is a change of coordinates for which

$$f(x, \theta) = g(y(x, \theta), \psi(\theta)) + \epsilon(\theta) \tag{12}$$

where $\psi(\theta)$ are the relevant parameters of the normal form [2]. By constructing our landscape from these two catastrophes is that it inherits this universality property and that as we will see now, the position and stability of the critical points and their dependence on the parameters is transparent.

The critical points of the binary flip with cusp model, defined in Eq. 2 in the main text, are given by the zeros of $\dot{x}$ and $\dot{y}$.

First, let us focus on the zeros of the equation regarding the change in time of y, since it does not involve x. This equation involves the flow determined by the fold, where we have introduced an additional parameter M, which allows for control of the position of the critical points:

$$\dot{y} = y f_{\text{fold}}(y - M, c) = -y((y - M)^2 + c) \tag{13}$$

The possible zeros of $\dot{y}$ are $y_1^* = M + \sqrt{-c}$, $y_2^* = M - \sqrt{-c}$ and $y_3^* = 0$. Since we assume that the state variables are real, y_1^* or y_2^* will only exist if $c \leq 0$.

The stability of the points y_i^* will depend on the sign of $\frac{d}{dy}(\dot{y})$ evaluated at the corresponding equilibrium point. Fixing the parameter $M > 0$, Fig. SI3 shows the corresponding bifurcation diagram. Note that the value of the parameter M does not really change the stability of the system, qualitatively. It only affects the interval in which c can take values, and the coordinates of the equilibrium point. We have decided to take positive values of M, and $H(y)$ to be the Heaviside function described in the main text. If M was negative, one would just need to take a different step function which value is 1 for $y \in [0, \infty)$, and this would just flip the state space over the y-axis.

We are only interested in the fold bifurcation that happens when $c = 0$, and not in the transcritical bifurcation at $c = -M^2$. Therefore we will assume that

$$c > -M^2 \tag{14}$$

from this point onwards. The parameter M does not affect the stability or the bifurcation of the system, it only controls the position of the critical points on the y axes and constrains the value of the parameter c. This is why we will not consider it as a control parameter in what follows. Summing up, depending on the value of c, Eq. 13 has one ($c > 0$), two ($c = 0$) or three ($c < 0$) equilibria.

Now let us consider the complete system described in Eq. 2. of the main text, and study the x-coordinates. In order to find the equilibrium points of the whole system we need to find the zeros of both equations. For $\dot{y} = 0$, as we saw before, the equation will have one, two or three zeros depending on the value of c. Let us now study the zeros of $\dot{x}$ in the case when $c < 0$, as the two other cases follow a similar logic.

When $c < 0$, as we saw earlier, $\dot{y} = 0$ has three possible roots: $y_1^* = M + \sqrt{-c}$, $y_2^* = M - \sqrt{-c}$ and $y_3^* = 0$. Let us show their corresponding x-coordinates:

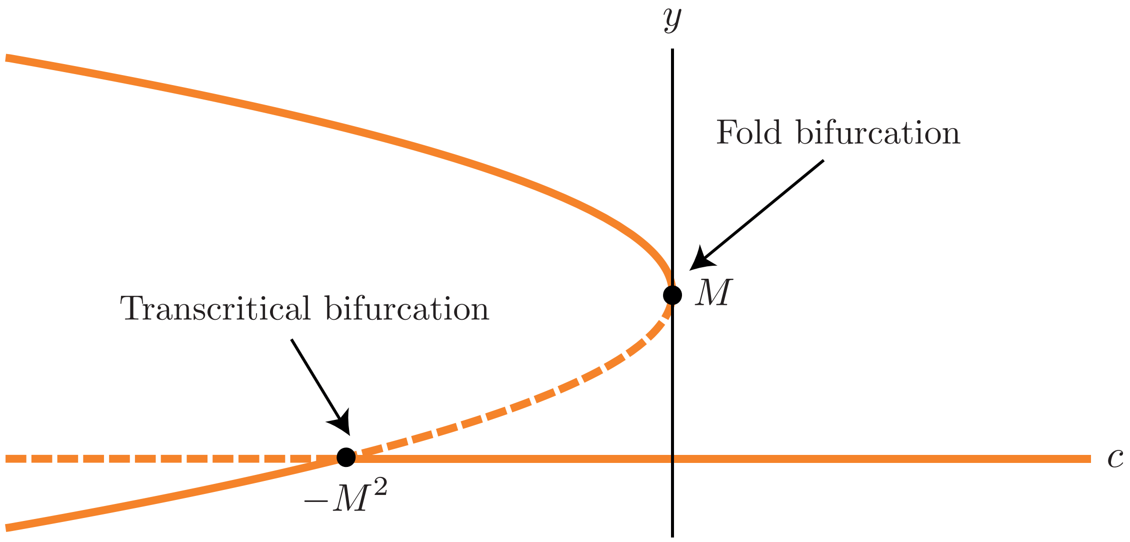

Figure SI3: Bifurcation diagram of Eq. 13. With $M > 0$ fixed, the orange lines show the equilibria of the system for different values of c. Continuous lines represent stable equilibria and dashed lines represent unstable equilibria.

- If $y = y_1^* = M + \sqrt{-c}$, since $H(-(M + \sqrt{-c})) = 0$,

$$\dot{x} = H(-(M + \sqrt{-c}))f_{\text{cusp}}(x, a, b) - (1 - H(-(M + \sqrt{-c})))x = -x = 0 \Leftrightarrow x = 0.$$

  Therefore there is an equilibrium point at $(0, M + \sqrt{-c})$.

- Also, if $y = y_2^* = M - \sqrt{-c}$, since $H(-(M - \sqrt{-c})) = 0$,

$$\dot{x} = H(-(M - \sqrt{-c}))f_{\text{cusp}}(x, a, b) - (1 - H(-(M - \sqrt{-c})))x = -x = 0 \Leftrightarrow x = 0.$$

  Hence, there is another equilibrium point at $(0, M - \sqrt{-c})$.

- And if $y = y_3^* = 0$, since $H(0) = 1$,

$$\frac{dx}{dt} = H(0)f_{\text{cusp}}(x, a, b) - (1 - H(0))x = f_{\text{cusp}}(x, a, b) = 0.$$

  Therefore the values for the x-coordinates, in this case, correspond to the equilibria of a cusp catastrophe with parameters a and b. Depending on the value of the discriminant $\Delta = 8a^3 + 27b^2$, the equation will have one ($\Delta > 0$), two ($\Delta = 0$) or three ($\Delta < 0$) real roots. This means that, depending on the value of Δ, there will be one, two or three equilibria on the x-axis.

The stability of these points can be checked by looking at the eigenvalues of the jacobian matrix:

$$J_{(a,b,c)}(x^*, y^*) = \begin{pmatrix} \frac{\partial f_1(x,y,a,b,c,M)}{\partial x} & \frac{\partial f_1(x,y,a,b,c,M)}{\partial y} \\ \frac{\partial f_2(x,y,a,b,c,M)}{\partial x} & \frac{\partial f_2(x,y,a,b,c,M)}{\partial y} \end{pmatrix}\Bigg|_{(x^*,y^*)} \tag{15}$$

where (x^*, y^*) is an equilibrium of the system in Eq. 2 in the main text, with parameters a, b, c, M. Since $\frac{\partial f_2(x,y,a,b,c,M)}{\partial x} = 0$, for all values of x, y, a, b, c, M, the Jacobian matrix is upper triangular and the eigenvalues are given by the elements on the diagonal.

In fact, the point $(0, M + \sqrt{c})$ is an attractor (which will represent tertiary fate), $(0, M - \sqrt{c})$ is a saddle point and the stability of the critical points on the x-axis depends on the value of the discriminant. Fig. SI4 shows the possible configurations of the equilibrium points in the state space.

When $c = 0$, it follows that there is only one (degenerate) critical point at $(0, M)$, and the critical points on the x-axis depend on the discriminant Δ and b, exactly as shown before. And finally, if $c > 0$, the tertiary fate has bifurcated away and the equilibria at the x-axis are the only ones that remain, which again depend on Δ and b.

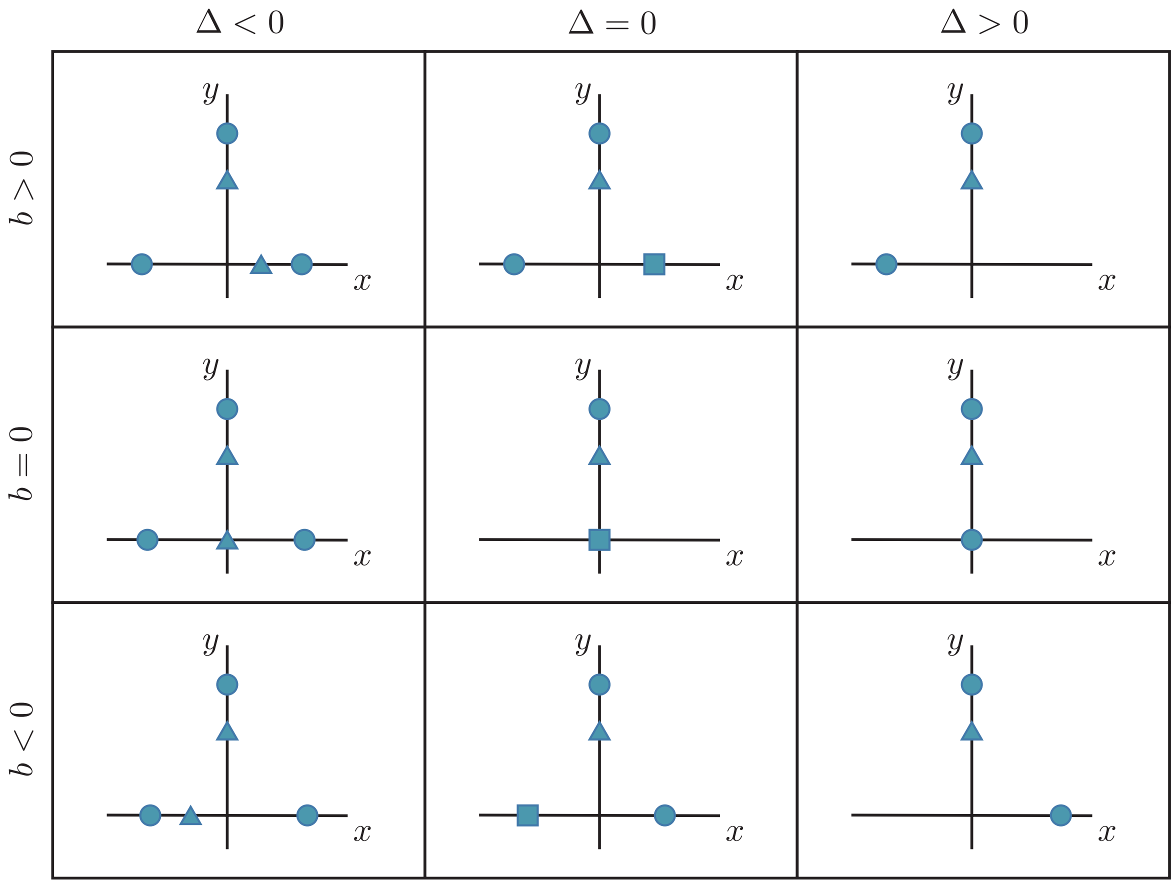

Figure SI4: Sketch of the equilibria of the system for $-M^2 < c < 0$ depending on the values of b and Δ. Stable equilibria are represented by circles. Saddles are represented by triangles. Degenerate steady states are represented by squares.

Summing up, the system can have from 5 to 1 equilibrium points in the state space depending on the values of the parameters a, b, c. There is a fold bifurcation point on the y-axis controlled by the parameter c, and a cusp bifurcation on the x-axis controlled by the parameters a and b.

2.2 Bifurcation set

We well now study the bifurcation set of the system by taking advantage of catastrophe theory. The catastrophe manifold of the system, $\mathcal{M}$, is the set of points $(x, y, a, b, c) \in \mathbb{R}^5$ such that (x, y) is an equilibrium point for the system in Eq. 2 for parameter values a, b and c. In other words,

$$\mathcal{M} = \{(x, y, a, b, c) \in \mathbb{R}^5 : f_1(x, y, a, b, c) = f_2(x, y, a, b, c) = 0\}.$$

Here we will show that $\mathcal{M}$ is a manifold that can be written as the union of two sets, one that corresponds to the fold bifurcation and another one that corresponds to the cusp bifurcation. Indeed, $(x, y, a, b, c) \in \mathcal{M}$ if and only if

$$f_1(x, y, a, b, c) = H(-y)(-4x^3 - 2ax - b) - (1 - H(-y))x = 0 \tag{16a}$$

and

$$f_2(x, y, a, b, c) = -y((y - M)^2 + c) = 0. \tag{16b}$$

From Eq. 16b we have that $y = 0$ or $y = M \pm \sqrt{-c}$. Now, if $y = 0$, from Eq. 16a:

$$-4x^3 - 2ax - b = 0 \iff b = -4x^3 - 2ax.$$

And if $y = M \pm \sqrt{-c}$, from Eq. 16a, x must be 0.

We can then express $\mathcal{M}$ as the disjoint union of two three-dimensional manifolds, $\mathcal{M}_1$ and $\mathcal{M}_2$, where:

$$\mathcal{M}_1 = \{(x, 0, a, -4x^3 - 2ax, c) \in \mathbb{R}^5 : x, a, c \in \mathbb{R}\} \quad (17a)$$

$$\mathcal{M}_2 = \{(0, y, a, b, -(y - M)^2) \in \mathbb{R}^5 : a, b \in \mathbb{R}, y \in (0, 2M)\} \quad (17b)$$

Therefore $\mathcal{M} = \mathcal{M}_1 \cup \mathcal{M}_2$ is a three-dimensional manifold in $\mathbb{R}^5$.

In order to visualise $\mathcal{M}$ we can plot the intersection with the three-dimensional space

$$\mathcal{N}_{(a_0,b_0)} = \{(x, y, a, b, c) \in \mathbb{R}^5 : a = a_0, b = b_0\}$$

to see how the equilibrium points change when c changes (see Fig. SI5).

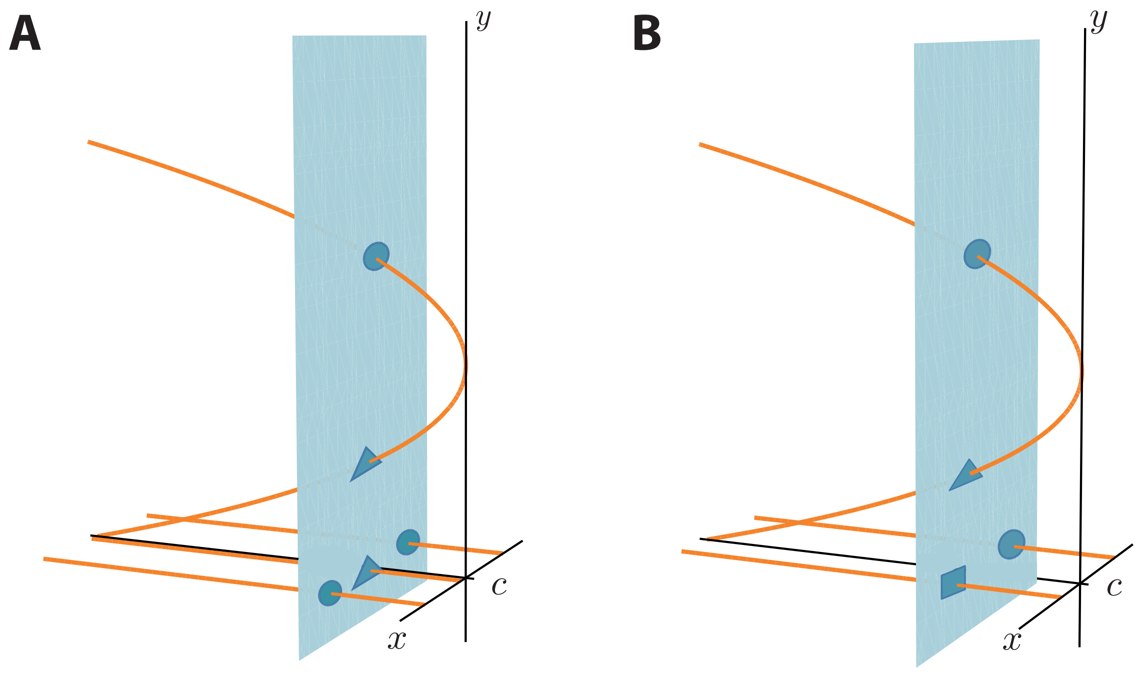

Figure SI5: Visualisation of the catastrophe manifold of the system in Eq. 2 of the main text.In orange, $\mathcal{M} \cap \mathcal{N}_{(a_0,b_0)}$ for different values of a_0, b_0. The plane π in cyan is obtained by fixing $c = c_0 < 0$, in other words, $\pi = \{(x, y, c) \in \mathbb{R}^3 : c = c_0\}$. The intersection of π and $\mathcal{M} \cap \mathcal{N}_{(a_0,b_0)}$ gives the equilibria of the system for parameters $a = a_0, b = b_0, c = c_0$. If sliding the plane π along the c axis, the equilibria on the y axis will move along the parabola. As before, circles represent stable equilibria, triangles represent saddles and squares represent degenerate points.

The **bifurcation set** is the set of values of the parameters at which bifurcations happen, i.e. generally the parameter values at which an attractor and a saddle collide and disappear. As mentioned in the main text, this set lets us characterise regions in the parameter space of common stability. By understanding the bifurcation set of the system, we can predict which equilibrium points will be present in the state space when knowing the values of the control parameters.

We first find an expression for the **singularity set** of the system, $\mathcal{S}$, defined in earlier, which is given by:

$$\mathcal{S} = \{(x, y, a, b, c) \in \mathbb{R}^5 : det(J_{(a,b,c)}(x, y)) = 0\}, \quad (18)$$

which is the subset of degenerate points of $\mathcal{M}$.

Let us denote $\frac{\partial f_1}{\partial x}$ by $D_x f_1$ and $\frac{\partial f_2}{\partial y}$ by $D_y f_2$. Since $det(J_{(a,b,c)}(x, y)) = D_x f_1 D_y f_2$, as explained earlier,

$$det(J_{(a,b,c)}(x, y)) = 0 \iff \begin{cases} D_x f_1(x, y, a, b, c) = H(-y)(-12x^2 - 2a) - (1 - H(-y)) = 0 \\ \quad \text{or} \\ D_y f_2(x, y, a, b, c) = -((y - M)^2 + c) - 2y(y - M) = 0 \end{cases}$$

Therefore we can write $\mathcal{S} = \mathcal{S}_1 \cup \mathcal{S}_2$ where

$$\mathcal{S}_1 = \mathcal{M} \cap \{(x, y, a, b, c) \in \mathbb{R}^5 : D_x f_1(x, y, a, b, c) = 0\} \tag{19}$$

and

$$\mathcal{S}_2 = \mathcal{M} \cap \{(x, y, a, b, c) \in \mathbb{R}^5 : D_y f_2(x, y, a, b, c) = 0\}. \tag{20}$$

Let us understand the geometry of these sets. The set $\mathcal{S}_1$ can be written as

$$\mathcal{S}_1 = (\mathcal{M}_1 \cup \mathcal{M}_2) \cap \{(x, y, a, b, c) \in \mathbb{R}^5 : D_x f_1(x, y, a, b, c) = 0\},$$

where $\mathcal{M}_1$ and $\mathcal{M}_2$ are defined in Eqs. 17a and 17b.

Applying the distributive property of the intersection over the union,

$$\mathcal{M}_1 \cap \{(x, y, a, b, c) \in \mathbb{R}^5 : D_x f_1(x, y, a, b, c) = 0\} = \{(x, 0, -6x^2, 8x^3, c) : x, y, c \in \mathbb{R}\},$$

and

$$\mathcal{M}_2 \cap \{(x, y, a, b, c) \in \mathbb{R}^5 : D_x f_1(x, y, a, b, c) = 0\} = \emptyset.$$

Therefore,

$$\mathcal{S}_1 = \{(x, 0, -6x^2, 8x^3, c) : x, y, c \in \mathbb{R}\}, \tag{21}$$

which corresponds to a cusp for every value of c (See Eq. 10).

On the other hand, the set $\mathcal{S}_2$ can be written as

$$\mathcal{S}_2 = (\mathcal{M}_1 \cup \mathcal{M}_2) \cap \{(x, y, a, b, c) \in \mathbb{R}^5 : D_y f_2(x, y, a, b, c) = 0\}.$$

Again, applying the distributive property of the intersection over the union,

$$\mathcal{M}_1 \cap \{(x, y, a, b, c) \in \mathbb{R}^5 : D_y f_2(x, y, a, b, c) = 0\} = \{(x, 0, a, -4x^3 - 2ax, -M^2) : a, c \in \mathbb{R}\}.$$

But, since we are only taking into account values of $c > -M^2$, we don't consider this set.

Moreover,

$$\begin{aligned}\mathcal{M}_2 \cap \{(x, y, a, b, c) \in \mathbb{R}^5 : D_y f_2(x, y, a, b, c) = 0\} &= \\ &= \{(0, 0, a, b, -M^2), a, b \in \mathbb{R}\} \cup \{(0, M, a, b, 0), a, b \in \mathbb{R}\},\end{aligned}$$

where, again, we do not consider the first subset since $c > -M^2$. Therefore

$$\mathcal{S}_2 = \{(0, M, a, b, 0), a, b \in \mathbb{R}\}, \tag{22}$$

which is a plane on the parameter space.

From Eqs. 21 and 22 we can write the singularity set, $\mathcal{S}$, of the system as:

$$\mathcal{S} = \{(x, 0, -6x^2, 8x^3, c) : x, y, c \in \mathbb{R}\} \cup \{(0, M, a, b, 0), a, b \in \mathbb{R}\} \tag{23}$$

Finally, projecting $\mathcal{S}$ into the control space we obtain an expression for the bifurcation set, $\mathcal{B}$:

$$\mathcal{B} = \mathcal{B}_1 \cup \mathcal{B}_2 = \{(a, b, c) \in \mathbb{R}^3 : 8a^3 + 27b^2 = 0\} \cup \{(a, b, 0) : a, b \in \mathbb{R}\} \tag{24}$$

which is a two-dimensional set in the three-dimensional control space (see Fig. SI6).

Fig. SI7 gives a description of the different landscapes in the state space depending on the values of the control parameters (a, b, c). We can see that crossing the orange plane in Fig. SI7C-E would bifurcate the points $(0, M - \sqrt{-c})$ and $(0, M + \sqrt{-c})$ and crossing the purple surface in Fig. SI7A-C and Fig. SI7E-G would bifurcate the points on the x-axis.

We have now obtained a map between the parameter space and the state space. Given a parameter value we know the exact topology of the state space. This gives us a description of how the topology of the space changes as we vary the parameters and therefore helps us to define the dynamical system that will describe the state of each VPC.

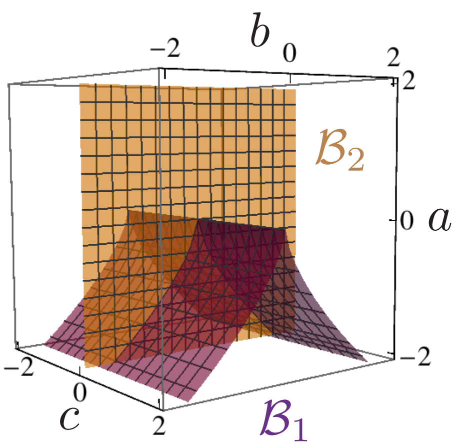

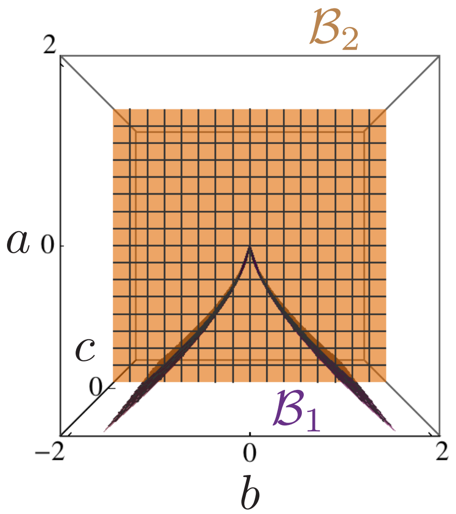

Figure SI6: Plots showing different views of the bifurcation set given in Eq. 24. The set $\mathcal{B}_1$ corresponds to the purple cuspoidal cylinder, while the set $\mathcal{B}_2$ corresponds to the orange plane. For every value of c the system has a cusp bifurcation set with a, b as parameters.Also, for any value of a, b, the points $(0, M - \sqrt{-c})$ and $(0, M + \sqrt{-c})$ bifurcate when $c = 0$, hence the plane.

2.2.1 Non-genericity of flip endpoint

We note that one aspect of our landscape is not generic. In a generic system the flip curve, the curve in the parameter space where the behavior described in Fig 1D happens (a heteroclinic connection between the two saddle points), would end not at the cusp point in parameter space (where $\Delta = b = 0$) but close to the cusp point at a point P on one of the fold lines adjoining the cusp (where $\Delta = 0$ but $b \neq 0$). For our system, the flip line starts at the cusp. This non-genericity is not important in this instance, in that a small deviation from the non-generic system will cause a small change in the point P and this does not affect our fitting.

2.3 Dependence upon the morphogens

In the previous section we have defined a dynamical system with a two-dimensional state space and a three-dimensional control space, and we have classified the possible landscapes that can appear in the state space. The goal is to take advantage of this dynamical system to define a system of differential equations such that its solution is a description of the state of each VPC during the process of differentiation.

As described in the main text, this process is controlled by two signalling pathways: induction from the anchor cell by EGF signal and lateral signalling through Notch. In consequence, the dynamics of the real system are controlled by these two parameters: level of EGF that the cell is receiving (this parameter will be denoted by θ_E) and level of Notch that the cell is receiving (parameter that will be denoted by θ_N). This means that the topology of the state space in the mathematical model should be related to the parameters θ_E and θ_N. In other words, the parameters a, b, c should be written as functions of θ_E and θ_N.

θ_E and θ_N can be regarded as a coordinate basis that generates a two-dimensional space. Let us call it the **signal space**. The coordinates of a point in that space will represent the values of the EGF and Notch signal that a cell receives.

Our goal is to find a correspondence between the signal space and the state space. That is to say that we aim to find which topology in the state space will correspond to which point in the signal space. And we can achieve this by finding a transformation from the (θ_E, θ_N) coordinate system to the (a, b, c) (Fig. SI8), which in turns determines the flow in the state space.

For simplicity, we will focus on affine transformations that map the signal space into the control space. This affine transformation, T, will be an embedding from the affine space $\mathbb{R}^2$ to the affine space $\mathbb{R}^3$, that maps the signal space into a plane in $\mathbb{R}^3$. These transformations will be characterised

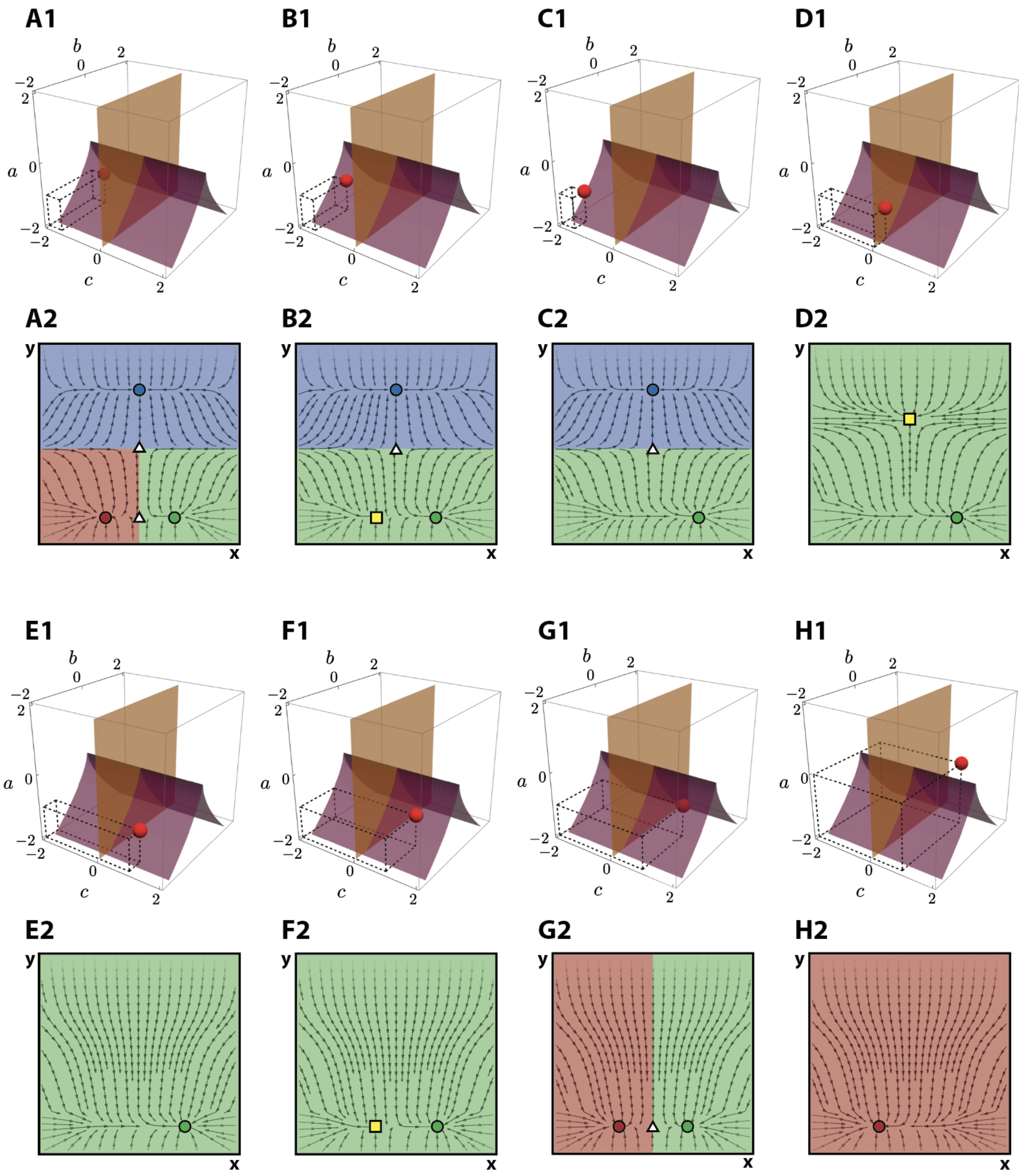

Figure SI7: Description of the flows defined by values the control parameters (a, b, c) positioned in the different regions of the control space defined by the bifurcation set. (A) $a = -1, b = 0, c = -1.5$ (B) $a = -1, b = -\sqrt{8/27}, c = -1.5$ (C) $a = -1, b = -1.5, c = -1.5$ (D) $a = -1, b = -1.5, c = 0$ (E) $a = -1, b = -1.5, c = 1$ (F) $a = -1, b = -\sqrt{8/27}, c = 1$ (G) $a = -1, b = 0, c = 1$ (H) $a = 0, b = 1, c = 1$

by the intersection of the planes that they define and the bifurcation set $\mathcal{B}$, since that will determine the possible dynamics of the system.

These affine transformations will have the form:

$$T : \mathbb{R}^2 \longrightarrow \mathbb{R}^3$$
$$X = (\theta_\mathrm{E}, \theta_\mathrm{N}) \mapsto T(X) = \begin{pmatrix} a \\ b \\ c \end{pmatrix} = \begin{pmatrix} m_{11} & m_{12} \\ m_{21} & m_{22} \\ m_{31} & m_{32} \end{pmatrix} \begin{pmatrix} s\theta_\mathrm{E} \\ l\theta_\mathrm{N} \end{pmatrix} + \begin{pmatrix} q_1 \\ q_2 \\ q_3 \end{pmatrix} \tag{25}$$

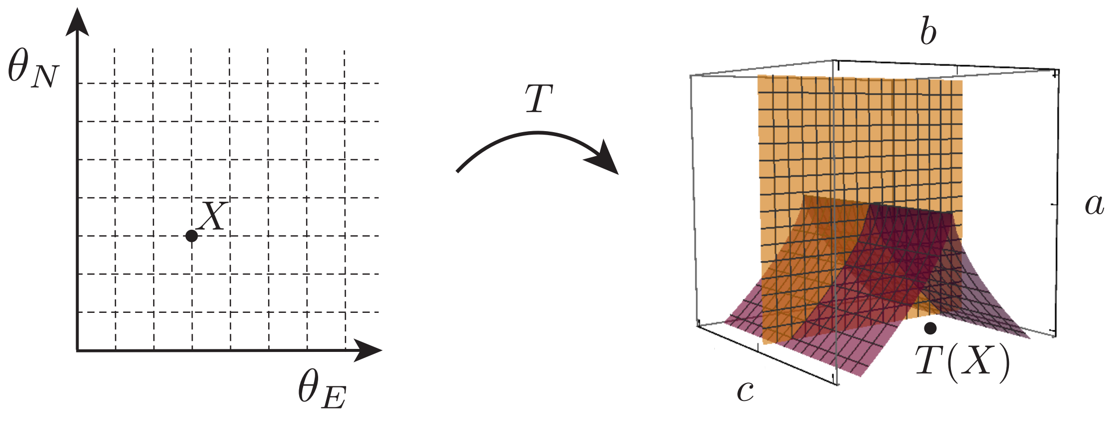

Figure SI8: Change of coordinates from the signal space to the control space. A transformation T will map a point X in the signal space (on the left) to a point $T(X)$ in the control space (on the right).

where m_{ij} and q_i define the transformation, and $s > 0$ and $l > 0$ are scaling parameters. This transformation maps a point in the signal space to a point in the plane $\pi_T = \{(a,b,c) \in \mathbb{R}^3 : Aa + Bb + Cc = D\}$ in the control space where $A = m_{31}m_{22} - m_{21}m_{32}$, $B = m_{11}m_{32} - m_{31}m_{12}$, $C = m_{12}m_{21} - m_{11}m_{22}$ and $D = -Aq_1 - Bq_2 - Cq_3$.

This plane intersects the bifurcation set $\mathcal{B}$ in different subsets depending on the values of m_{ij}, and the origin of the $(\theta_{\mathrm{E}}, \theta_{\mathrm{N}})$ coordinate space in that plane will be determined by the parameters q_i (see Fig. SI9).

We will assume that

$$m_{11}^2 + m_{21}^2 + m_{31}^2 = 1, \tag{26}$$

$$m_{12}^2 + m_{22}^2 + m_{32}^2 = 1, \tag{27}$$

$$m_{11}m_{12} + m_{21}m_{22} + m_{31}m_{32} = 0, \tag{28}$$

i.e., T is conformal (it preserves angles).

We also assume that the origin of the $(\theta_{\mathrm{E}}, \theta_{\mathrm{N}})$ coordinate system is mapped to a point in which there is tristability, this is

$$\begin{cases} 8a(0,0)^3 + 27b(0,0)^2 = 8q_1^3 + 27q_2^2 < 0 \\ c(0,0) = q_3 < 0. \end{cases} \tag{29}$$

This is because of the definition of commitment of stem cells. Once a stem cell is committed, signals can be removed and the stem cell will not alter its fate. In the process of differentiation of the VPCs of a WT worm, they become specified into the three different fates (See Table 1 in the main text) [3]. If we imagine the signal history of a VPC as a path in the signal space, this means that after having received different signals during their development, if the signals are switched off and the path returns to the point $\theta_{\mathrm{E}} = \theta_{\mathrm{N}} = 0$, the three attractors must be present for each VPC to be able to specify to their corresponding WT fates (P4.p specifies into 3°, P5.p specifies into 2° and P6.p specifies into 1°).

We could constrain the transformation so that high θ_{E} pushes towards monostability of 1° fate (i.e. $m_{21} > 0$) and high θ_{N} pushes towards monostability of 2° fate (i.e. $m_{22} < 0$). However, we decide not to constraint the system so much, and see what the data fitting decides as the best strategy.

Finally, considering possible intersections between the plane π_T and the bifurcation set, we decide to only accept transformations such that $A \neq 0 \neq C$. This is not an important constraint, since the sets $A = 0$ or $C = 0$ in $\mathbb{R}^3$ have zero measure. Moreover, by taking $A = \varepsilon$ or $C = \varepsilon$ with ε very small, one could approximate these special cases. Making this restriction allows us to easily classify the types of transformation that can be allowed. With this in mind we assume that $A \neq 0 \neq C$ and, consequently, the transformations can be of two types: affine transformations such that $AC < 0$ (let us call them Type I) or affine transformations such that $AC > 0$ (let us call them Type II).

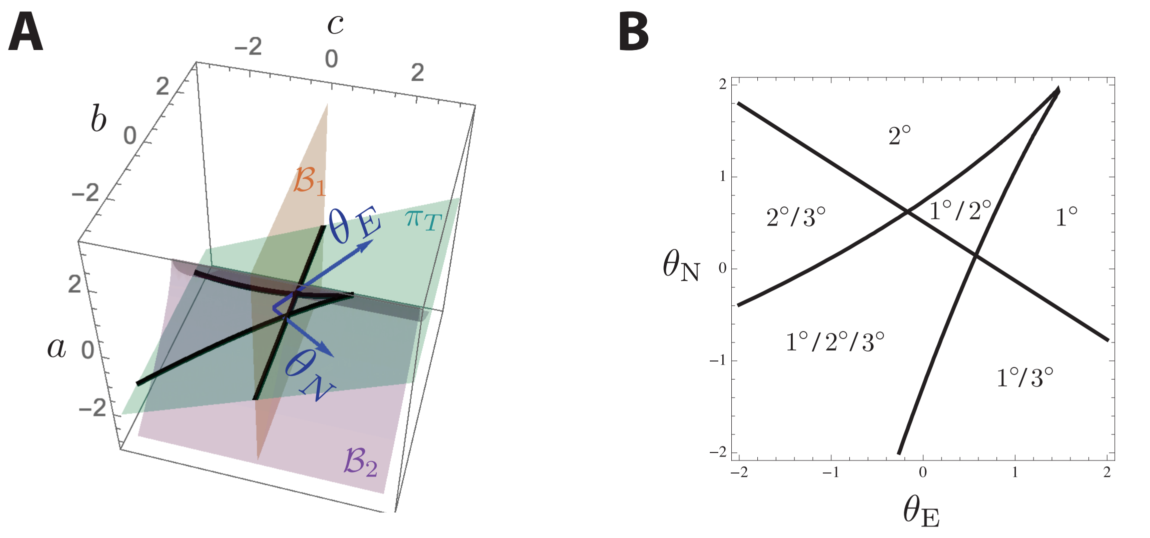

Figure SI9: An example of linear transformation T from the signal space to the control space. (A) The figure shows, in the control space, the plane π_T in light blue, the bifurcation set $\mathcal{B} = \mathcal{B}_1 \cup \mathcal{B}_2$ in orange and purple, the intersection $\pi_T \cap \mathcal{B}$ in thick black lines and the transformation of the (θ_E, θ_N) axis in dark blue. For simplicity, we have named this new coordinate system in the control space as the original in the signal space. (B) Signal space. The thick black lines correspond to the set $T^{-1}(\pi_T \cap (\mathcal{B}_1 \cup \mathcal{B}_2))$. These lines define regions in the signal space that will generate different configurations of attractors in the state space. In each region, the fates which attractors will be present for a value of signals in the corresponding region are written. For $\theta_E = 0 = \theta_N$, the system will contain the three attractors (one for each fate). For a *medium* value for θ_E and only the attractors corresponding to 1° and 2° fates will be present. For high values for θ_E and low values of only the attractor corresponding to 1° fate will be present. And, for low values for θ_E and high values of , only the attractor corresponding to 2° fate will be present.

2.3.1 Type I transformations

Type I transformations are such that π_T intersects the plane $b = 0$ on a line $a = (D - Cc)/A$, and the gradient $-C/A$ is positive (see Fig. SI10).

Since a tristable region needs to be present in the intersection $\pi_T \cap \mathcal{B}$, this forces

$$D/A < 0. \tag{30}$$

On the other hand, because of the experimental results for the Notch null with 2×EGF mutant (See experiment (5) in Table 1 in the main text), and *lin* − 12 gain-of-function experiments in [4] (which can be considered as θ_N high), we constrain the system so that θ_E high or θ_N high drive the system out of the tristable region. Therefore,

$$m_{31}, m_{32} > 0 \quad \text{and} \quad m_{11}, m_{12} > 0. \tag{31}$$

Examples of Type I transformations that satisfy constraints in Eqs. 30 and 31 are given in Figures SI11.

Eqs. 26, 27, 28 and 31 again allow us to rewrite the parameters m_{31}, m_{22} and m_{32} of the transformation as functions of the other parameters, lowering the levels of freedom. In particular:

$$m_{31} = \sqrt{1 - m_{11}^2 - m_{21}^2} \tag{32}$$

which let us write m_{22} and m_{32} as the solutions of the system

$$\begin{cases} m_{12}^2 + m_{22}^2 + m_{32}^2 = 1 \\ m_{11}m_{12} + m_{21}m_{22} + m_{31}m_{32} = 0 \end{cases} \tag{33}$$

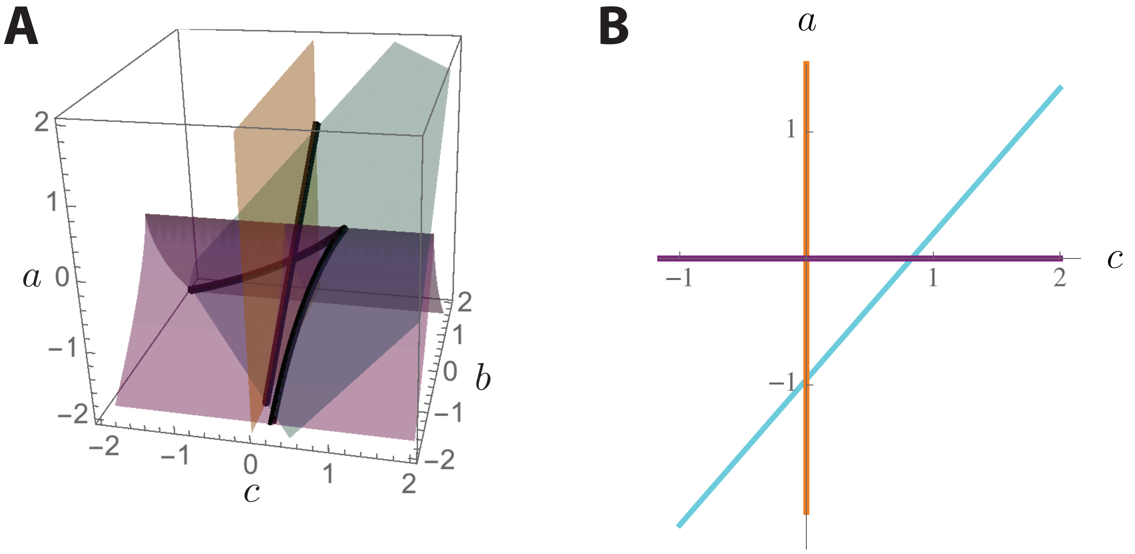

Figure SI10: Example of the intersection $\pi_T \cap \mathcal{B}$ for a T such that $AC < 0$. (A) Intersection of the plane $\pi_T = \{(a, b, c) \in \mathbb{R}^3 : a - b - c = -1\}$ and the bifurcation set $\mathcal{B}$. The set $\mathcal{B}_1$ is plotted in orange, the set $\mathcal{B}_2$ is plotted in purple and the plane π_T is plotted in cyan. The intersection is marked by thick black lines. (B) In orange, the intersection $\mathcal{B}_1 \cap \{(a, 0, c) \in \mathbb{R}^3\}$. In purple, the intersection $\mathcal{B}_2 \cap \{(a, 0, c) \in \mathbb{R}^3\}$. In cyan, the intersection $\pi_T cap\{(a, 0, c) \in \mathbb{R}^3\}$. As we can see, the line has positive gradient.

2.3.2 Type II transformations

On the other hand, Type II transformations are such that π_T intersects the plane $b = 0$ in the control space on a line $a = (D - Cc)/A$, and the gradient $-C/A$ is negative (see Fig. SI12).

Since a tristable region needs to be present in the intersection $\pi_T \cap \mathcal{B}$, this forces

$$D/A < 0. \tag{34}$$

On the other hand, because of the experimental results for the Notch null with 2×EGF mutant (See experiment (5) in Table 1 in the main text), and $lin - 12$ gain-of-function experiments in [4] (which can be considered as high), we constrain the system so that θ_{E} high or high drive the system out of the tristable region. Therefore,

$$m_{31}, m_{32} > 0 \quad \text{and} \quad m_{11}, m_{12} < 0. \tag{35}$$

Examples of Type I transformations that satisfy the constraints in Eqs. 34 and 35 are given in Fig. SI13.

Eqs. 26, 27, 28 and 35 allow us to rewrite three parameters of the transformation as functions of the other parameters, lowering the levels of freedom. In particular:

$$m_{31} = \sqrt{1 - m_{11}^2 - m_{21}^2}$$

which let us write m_{22} and m_{32} as the solutions of the system

$$\begin{cases} m_{12}^2 + m_{22}^2 + m_{32}^2 = 1 \\ m_{11}m_{12} + m_{21}m_{22} + m_{31}m_{32} = 0 \end{cases}$$

Here we focus on Type I transformations, as they fit the data better. Type II transformations failed to reproduce some EGF overexpression data.

Considering the system defined in Eq. 2 in the main text, and the linear transformations with the corresponding constraints, we can now present the proposed model that will describe the process of differentiation of the VPCs.

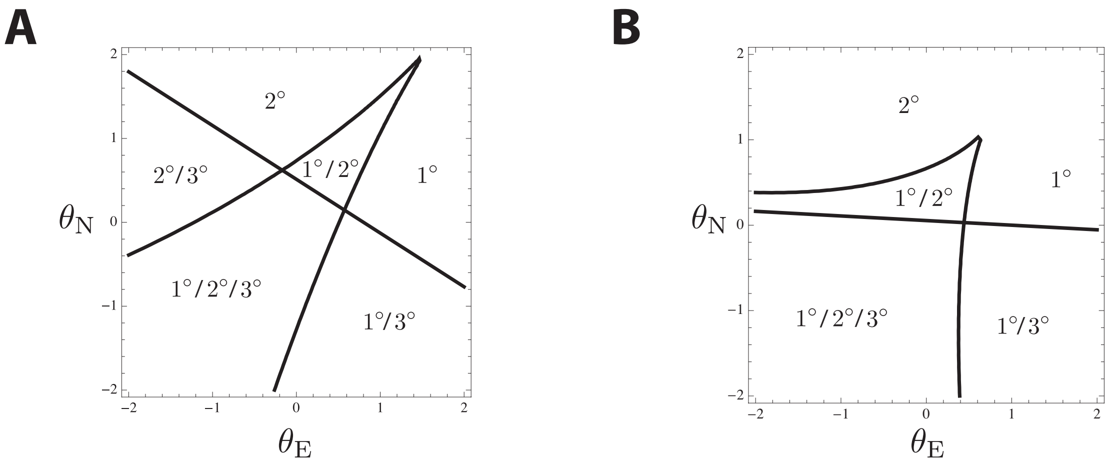

Figure SI11: Two examples of bifurcation sets in the signal space given by the intersections $T^{-1}(\pi_T \cap \mathcal{B})$ for two different Ts of Type I satisfiying constraints in Eqs. 30 and 31. As in Fig. SI9, different regions are labeled with the corresponding attractors.

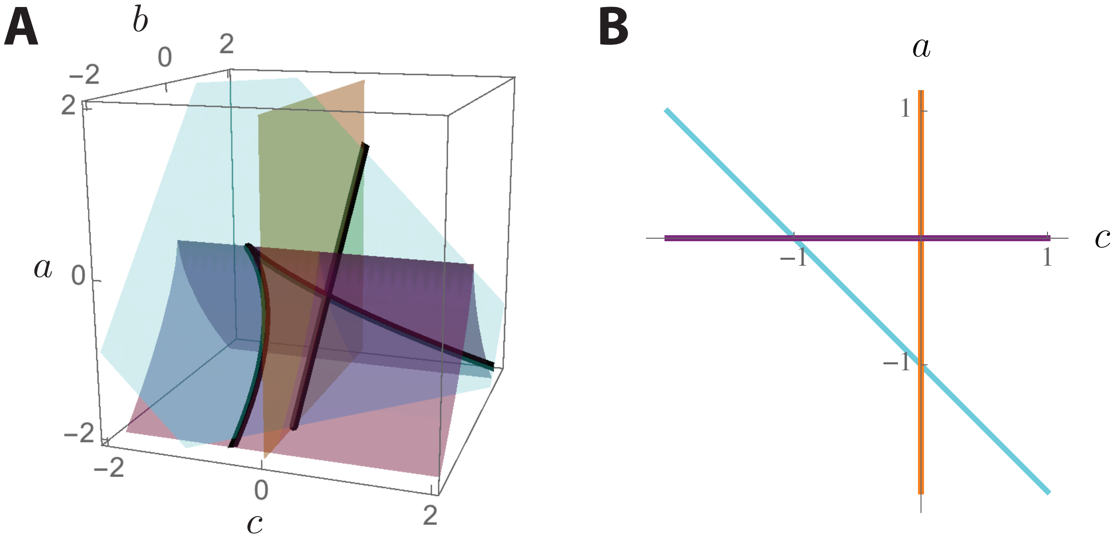

Figure SI12: Example of the intersection $\pi_T \cap \mathcal{B}$ for a T such that $AC > 0$. (A) Intersection of the plane $\pi_T = \{(a, b, c) \in \mathbb{R}^3 : a+b+c = -1\}$ and the bifurcation set $\mathcal{B}$. The set $\mathcal{B}_1$ is plotted in orange, the set $\mathcal{B}_2$ is plotted in purple and the plane π_T is plotted in cyan. The intersection is marked by thick black lines. (B) In orange, the intersection $\mathcal{B}_1 \cap \{(a, 0, c) \in \mathbb{R}^3\}$. In purple, the intersection $\mathcal{B}_2 \cap \{(a, 0, c) \in \mathbb{R}^3\}$. In cyan, the intersection $\pi_T \cap \{(a, 0, c) \in \mathbb{R}^3\}$. As we can see, the line has negative gradient.

2.4 Model implementation and numerical simulations

So far we have developed a model that describes the process of differentiation of a single VPC in time, given the signals EGF and Notch. However, the biological system is formed by 6 VPCs (P3-8.p), that interact with each other. However, taking into account that P3.p normally fuses with the hypodermis and that the pattern is usually symmetric around the AC, we will model the development of 3 VPCs: P4.p, P5.p and P6.p, where P6.p is the closest to the AC. This means that the mathematical model will be comprised of three trajectories, each corresponding to a VPC, moving on their corresponding landscapes which shapes will depend on the signals that each cell receives. Our proposed model is therefore described by the following set of equations, with (x_1, y_1) being the coordinates representing the state of differentiation of P4.p, (x_2, y_2) the coordinates representing the state of P5.p and (x_3, y_3)

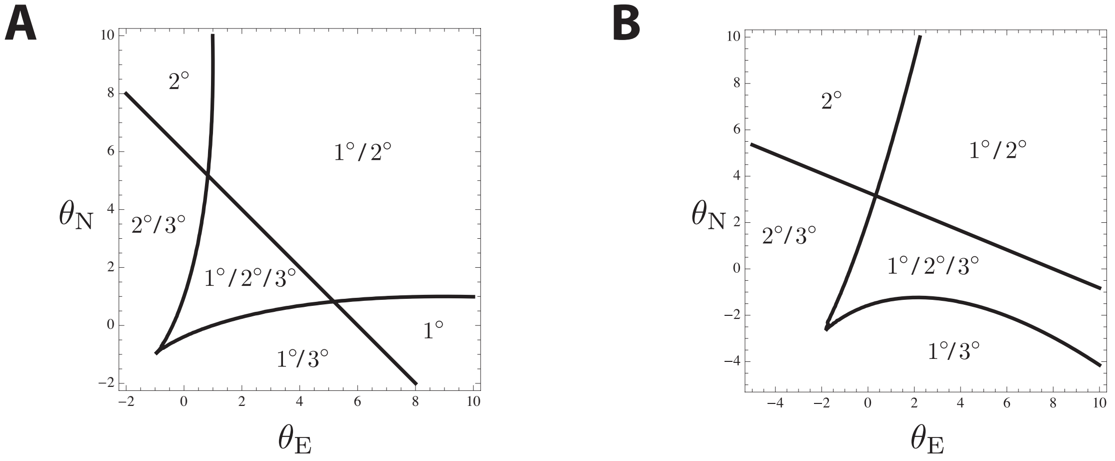

Figure SI13: Two examples of bifurcation sets in the signal space given by the intersections $T^{-1}(\pi_T \cap \mathcal{B})$ for two different Ts of Type I satisfiying constraints in Eqs. 34 and 35. As in Fig. SI9, different regions are labelled with the corresponding attractors.

the coordinates representing the state of P6.p:

$$\begin{cases} \frac{dx_i}{dt} = (1/\tau)[\chi(y_i) f_{\text{cusp}}(x_i, a_i, b_i) - (1 - \chi(y_i))x_i] + \eta_{2i-1}(t) \\ \frac{dy_i}{dt} = (1/\tau)[y_i f_{\text{fold}}(y_i, c_i, M)] + \eta_{2i}(t) \end{cases} \tag{36}$$

where, $1/\tau$ is a time constant, $f_{\text{cusp}}(x, a, b) = -4x^3 - 2ax - b$, $f_{\text{fold}}(y, c, M) = -(y - M)^2 - c$ are the functions explained above and, in order to work with smooth functions, we changed $H(-y)$ to $\chi(y)$, a sigmoidal function of the form:

$$\chi(y) = \frac{1 + \tanh\left(H\left(-y + rM\right)\right)}{2}, \tag{37}$$

where we choose $H = 25$ and $r = \frac{\sqrt{2}-1}{2\sqrt{2}}$. A white noise η_i is also added to the system, with variance given by a parameter $2\sigma_{dif}$, to account for intrinsic variability:

$$\langle \eta_i(t)\eta_j(t')\rangle = 2\sigma_{dif}\delta_{ij}\delta(t - t'). \tag{38}$$

The values of the control parameters for each cell are given by:

$$\begin{pmatrix} a_i \\ b_i \\ c_i \end{pmatrix} = T(\theta_{E,i}, \theta_{N,i}) = \begin{pmatrix} m_{11} & m_{12} \\ m_{21} & m_{22} \\ m_{31} & m_{32} \end{pmatrix} \begin{pmatrix} s\theta_{E,i} \\ l\theta_{N,i} \end{pmatrix} + \begin{pmatrix} q_1 \\ q_2 \\ q_3 \end{pmatrix}, \tag{39}$$

where, as explained above, T is either of Type I, satisfying the constraints in Table SI1, where the definitions of A, C and D and the constraints were explained before.

Following the approach by [5, 6], we assume that the difference of EGF signal between consecutive cells is regulated by a scaling parameter γ, which derivation follows from the diffusion of EGF morphogen ($\gamma < 1$). However, we also model the increase of EGF signal in time as a monotonous increasing function $\sigma(t)$:

$$\theta_{\text{E}}^1 = \gamma^2\sigma(t), \quad \theta_{\text{E}}^2 = \gamma\sigma(t), \quad \theta_{\text{E}}^3 = \sigma(t), \tag{40}$$

where the parameter s is a scaling factor and the function $\sigma(t)$ is given by:

$$\sigma(t) = \frac{1 + \tanh(H_E t + M_E)}{2},$$

where H_E and M_E are parameters to be fitted.

We also define the parameter to be proportional to the sum of the autocrine Notch signal of the cell (signal produced by the cell itself) and the paracrine Notch signal received by the cell (produced

**Constraints**
$A \neq 0$
$C \neq 0$
$8q_1^3 + 27q_2^2 < 0$
$q_3 < 0$
$AD < 0$
$m_{31} = \sqrt{1 - m_{11}^2 - m_{21}^2}$
m_{22}, m_{32} solutions of $\begin{cases} m_{12}^2 + m_{22}^2 + m_{32}^2 = 1 \\ m_{11}m_{12} + m_{21}m_{22} + m_{31}m_{32} = 0 \end{cases}$
$m_{31}, m_{32} > 0$
$AC < 0$
$m_{11}, m_{12} > 0$

Table SI1: Table of mathematical constraints on the affine transformation T of Type I from the signal space to the control space.

by the neighboring cells). The autocrine signal is multiplied by a parameter α which parametrises the relative importance of autocrine and paracrine signalling. We also take into account that 1° fated cells downregulate Notch receptor lin-12, and therefore downregulate the Notch signal received, as suggested in [7]. This downregulation is related to the cell's production of Notch signalling, scaled by a parameter l_d which defines the strength of such downregulation. Therefore, we define the levels as

$$\theta_{\mathrm{N}}^1 = (1 - l_d L(x_1, y_1))(\alpha L(x_1, y_1) + L(x_2, y_2)), \tag{41}$$
$$\theta_{\mathrm{N}}^2 = (1 - l_d L(x_2, y_2))(L(x_1, y_1) + \alpha L(x_2, y_2) + L(x_3, y_3)), \tag{42}$$
$$\theta_{\mathrm{N}}^3 = (1 - l_d L(x_3, y_3))(\alpha L(x_3, y_3) + 2L(x_2, y_2)), \tag{43}$$

where the parameter l is a scaling factor. The function L represents the Notch signal emitted by a cell, which depends on the current state of the cell, and it is chosen as:

$$L(\boldsymbol{x}(t)) = \frac{1 + \tanh(n_0 + \boldsymbol{n}_1 \cdot \boldsymbol{x}(t))}{2}, \tag{44}$$

where n_0 and $\|\boldsymbol{n}_1\|$ are constants. L should be increasing as the state of the cell approaches the attractor corresponding to 1° fate, therefore we fit the y-coordinate of $\boldsymbol{n}$, $n_{1,y}$, and fix its x-coordinate to be $n_{1,x} = -\sqrt{1 - n_{1,y}^2}$, so that L increases as a cell moves toward the basin of attraction of 1° fate.

Taking into accounts the constraints of the linear transformation discussed above, the development of the VPCs is then defined by a 6-dimensional system of stochastic differential equations depending on the twenty parameters in Table SI2.

The model and following steps are implemented in Matlab, and the code is provided in the following github repository:

https://github.com/ecamacho90/VulvalDevelopment

2.4.1 Initial condition

In order to perform a simulation, first, it is necessary to choose an initial condition that will represent the state of the VPCs at the start of the experiment. We assume that the VPCs are initially equivalent and that their initial condition should not depend on the signals they will receive. Since in the absence of signals they take the 3° fate, we assume that their initial condition should lie in the basin of attraction of the attractor corresponding to the 3° fate.

	Parameter	Description
Landscape	$1/\tau$	Time constant
	M	Position of the saddle point between 3° fate and 1°-2° fates
	m_{11}	Linear transformation between signal space and control space
	m_{12}	Linear transformation between signal space and control space
	m_{21}	Linear transformation between signal space and control space
	q_1	Position origin signal space in control space, linear transformation
	q_2	Position origin signal space in control space, linear transformation
	q_3	Position origin signal space in control space, linear transformation
	s	EGF signal scaling constant
	l	Notch signal scaling constant
Signals	γ	EGF diffusion
	H_E	Gradual EGF increase in time
	M_E	Gradual EGF increase in time
	l_d	Stregnth of Notch inhibition in 1° fated cells
	α	Autocrine Notch signaling strength
	n_0	Notch signal sigmoidal function
	n_1	Notch signal sigmoidal function
	$n_{1,y}$	Notch signal sigmoidal function
	λ_E	Exponential decay of EGF
	λ_N	Exponential decay of Notch signal
	σ_{dif}	White noise variance

Table SI2: Table of model parameters. Table containing the landscape parameters that map the signaling values into the shape of the landscape and 15 signaling parameters that define the signaling profile that each cell is exposed to in time.

It is also reasonable to incorporate some variability in the initial state, so we decide to choose as initial condition of the full system, the stationary distribution around the equilibrium of the following system of SDEs

$$\begin{cases} \frac{dx}{dt} &= \frac{1}{\tau}[\chi(y)(-4x^3 - 2a_0x - b_0) - (1-\chi(y))x] + \eta_{01}(t) \\ \frac{dy}{dt} &= -\frac{1}{\tau}[y((y-M)^2 + c_0)] + \eta_{02}(t) \end{cases} \tag{45}$$

where $a_0 = q_1$, $b_0 = q_2$, $c_0 = q_3$ are obtained from Eq. 39 by setting $\theta_{\rm E} = 0$ and $\theta_{\rm N} = 0$ and η_{01} and η_{02} represent independent white noises with variance $2\sigma_{dif}$.

As initial condition of the system in Eq. 45 we choose a normal distribution centered at $(x(0), y(0)) = (0, M)$ with zero covariance matrix, to make sure that the initial condition stays in the basin of attraction of the tertiary fate.

We decide to approximate such stationary distribution numerically. In order to do that, we considered two approaches. As a first attempt, we approximated such distribution by running N simulations ($N = 100, 1000$) for a long enough period of time by taking advantage of the Euler-Maruyama method. The stationary distribution was approximated by the distribution of the end points of those N simulations. As a second approach, we approximated the solution of the SDE based on a similar method to derive the Linear Noise Approximation (LNA) [8, 9], and computed the solution for a long enough period of time ($t \in (0, 10)$). In order to get the same accuracy with the two methods, the first approach took twice as much time as the second approach, so we decided to use the second one.

2.4.2 Simulation procedure

Eq. 36 is solved by using Euler-Maruyama [10, 11, 12]. We draw N initial conditions from the initial distribution computed as we explained before and simulate N random walks with the Euler Maruyama method. The system in Eq. 36 is simulated from $t = 0$ to $t = 1$, with time step $dt = 0.005$, with signals active, to account for the competence period in which the VPCs respond to signals. Then, the system is continued from $t = 1$ to $t = 3$, with $dt = 0.005$, with signals off to account for the post-competence period. We consider this post-competence period because, in order for cells to be

specified, they should keep their fates even if they do not receive any signals. During this post-competence period we consider an exponential decay of the signals (we found anomalous solutions if we instantaneously set $\theta_{\rm E} = \theta_{\rm N} = 0$, and it is also more biologically reasonable). Therefore, during the post-competence period, we replace s and l by:

$$s^{PC}(t) = se^{-\lambda_E(t-t_1)},$$
$$l^{PC}(t) = le^{-\lambda_N(t-t_1)}.$$

The parameters λ_E and λ_N are fitted but constrained so that, at $t = 3$, $s^{PC}(3), l^{PC}(3) \approx 0$.

At time $t = 3$, for each one of the N simulations, the fates of the three VPCs are scored as will be explained in the following section. The simulated outcome is given by the proportion of times that each cell took each fate. Therefore, the simulated outcome can be sumarised in a matrix

$$\mathcal{D}^{sim} = \begin{pmatrix} p_{11}^{sim} & p_{12}^{sim} & p_{12}^{sim} & p_{14}^{sim} \\ p_{21}^{sim} & p_{22}^{sim} & p_{22}^{sim} & p_{24}^{sim} \\ p_{31}^{sim} & p_{32}^{sim} & p_{32}^{sim} & p_{34}^{sim} \end{pmatrix}$$

where for $j = 1, 2, 3$ the value p_{ij}^{sim} is the proportion of times, in the N simulations, that P$(i+3)$.p took fate $j°$. For $j = 4$ the value p_{ij}^{sim} is the proportion of times, in the 150 simulations, that P$(i+3)$.p could not be assigned a fate (See next subsection for more details). We checked the sensitivity of $\mathcal{D}^{sim}$ with respect to the number of simulations and we found that $N = 150$ gave best results.

2.4.3 Fate assignment

Given one simulation of the system, the goal is to compute the fate of each cell. As explained above, we score the fates at time $t = 3$. At that time, the signals are switched off for the three VPCs, so they lie on the same fixed flow defined by the system of equations:

$$\left\{ \begin{array}{lcl} \frac{dx}{dt} & = & \frac{1}{\tau}[\chi(y)(-4x^3 - 2a_{PC}x - b_{PC}) - (1-\chi(y))x] \\ \frac{dy}{dt} & = & -\frac{1}{\tau}[y((y-M)^2 + c_{PC})] \end{array} \right. \tag{46}$$

where $\chi(y)$ is defined in Eq. 37, $a_{PC} = q_1$, $b_{PC} = q_2$, $c_{PC} = q_3$, such that it contains the three attractors (one for each fate), as explained above.

Depending on the value of $b_{PC} = q_2$, the basins of attraction will look like the ones represented in Fig. SI14.

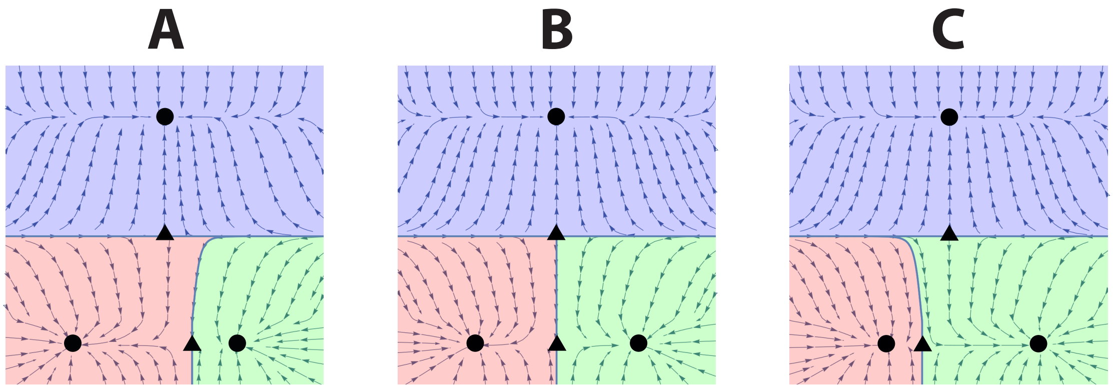

Figure SI14: Possible landscapes for post-competence period for different values of q_2, when the control parameters take values $a_{PC} = q_1$, $b_{PC} = q_2$, $c_{PC} = q_3$. Arrows represent the flow defined by the system in Eq. 46. Basins of attraction are coloured blue, green and red from attractors corresponding to the 3°, 2° and 1° fates, respectively.

In order to assign a fate to a VPC in one simulation, we find the basin of attraction in which the trajectory lies at time $t = 3$, and allocate the corresponding fate.

Let us call (x_i, y_i) coordinates of a cell at time $t = 3$ for a particular simulation. In order to find the basin of attraction in which it lies we do the following:

1. Suppose $y_i > M - \sqrt{-c}$. As we see in Fig. SI15, the basin of attraction corresponding to the 3° fate is the region of the state space defined by $\{(x, y) \in \mathbb{R}^2 : y > M - \sqrt{-c}\}$, where $M - \sqrt{-c}$ is the y-coordinate of the yellow saddle in Fig. SI15.

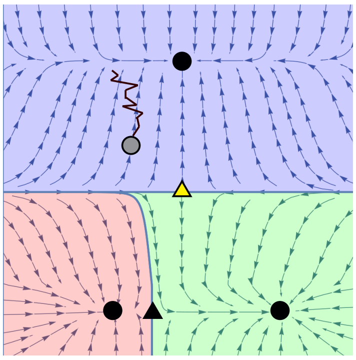

Figure SI15: Example of an assignment of the fate 3° to a VPC in a simulation. Arrows represent the flow defined by the system in Eq. 46. Basins of attraction are coloured blue, green and red from attractors corresponding to the 3°, 2° and 1° fates, respectively. The end point (gray circle) of a simulated trajectory of a VPC during the post-competence period (black path) lays in the basin of attraction of 3° fate (blue region).

   Therefore, if $y_i > M - \sqrt{-c}$, then we assign the 3° fate to this VPC in such a simulation.

2. If $y_i = M - \sqrt{-c}$, then the point lies on the boundary between two basins of attraction. We consider that we cannot assign a fate to the VPC in this case.

3. If $y_i < M - \sqrt{-c}$ then the point lies in the basin of attraction of either 1° or 2° fate.

   (a) If $q_2 = 0$, the saddle between the red and green regions in Fig. SI14 has coordinates $(0, 0)$ and it is half way between the attractors corresponding to fates 1° and 2°. In this case, the basin of attraction corresponding to 1° fate is defined by the set of points with $x < 0, y < M - \sqrt{-c}$. Moreover, the basin of attraction corresponding to the 2° fate is defined by the set of points given by $x > 0, y < M - \sqrt{-c}$. Consequently, we assign fate 1° (resp. 2°) to such a VPC if $x_i < 0$ (resp. $x_i > 0$).

   (b) If $q_2 \neq 0$, we observe that the stable manifold of the saddle between the red and green regions, which serves as boundary between the two basins of attraction, bends towards the closer of the two attractors (see Fig. SI14).
   Consider the case $q_2 < 0$ (similarly if $q_2 > 0$). In this case, the landscape would look like the leftmost landscape in Fig. SI14. Let $(x_1^*, 0)$ be the coordinates of the attractor corresponding to the 1° fate, $(x_2^*, 0)$ be the coordinates of the attractor corresponding to the 2° fate, $(x_s^*, 0)$ be the coordinates of the saddle determining the boundary between the basins of attraction of 1° and 2° fates, and $(0, M - \sqrt{-c_{PC}})$ be the coordinates of the saddle determining the basin of attraction of the 3° fate. We assign the fate as follows:

      i. If $(x_i, y_i) \in C_2 = \{(x, y) \in \mathbb{R}^2 : \|(x, y) - (x_2^*, 0)\| \leq R\|(x_s^*, 0) - (x_2^*, 0)\|$, i.e. (x_i, y_i) is in the dark green circle arround the attractor corresponding to 2° fate in Fig. SI16), we assign the fate 2°.

      ii. If $(x_i, y_i) \in C_1 = \{(x, y) \in \mathbb{R}^2 : \|(x, y) - (x_1^*, 0)\| \leq S \min\{\|(x_s^*, 0) - (x_1^*, 0)\|, M - \sqrt{-c_{PC}}\}$ i.e. (x_i, y_i) is in the dark red circle around the attractor corresponding to 1° fate in Fig. SI16), we assign fate 1°.

      iii. Otherwise, we solve the system of ordinary differential equations in Eq. 46 until the trajectory crosses either C_1 or C_2, assigning the corresponding fate.

   Where we take $R = 0.9$ and $S = 10^{-3}$.

2.4.4 Simulation of mutants

If we call

$$\boldsymbol{\theta}_{WT} = (1/\tau, H, M, m_{11}, m_{12}, m_{21}, q_1, q_2, q_3, s, \gamma, H_E, M_E, l, l_d, \alpha, n_0, n_1, n_{1,y}, \lambda_E, \lambda_N, \sigma_{dif}),$$

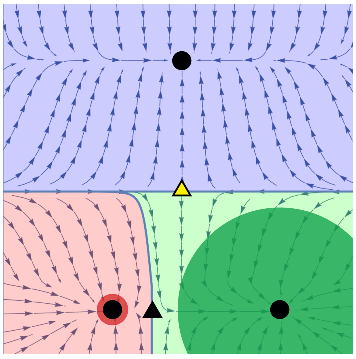

Figure SI16: Assignment of 1° or 2° fates when $q_2 < 0$. Arrows represent the flow defined by the system in Eq. 46. Basins of attraction are colored blue, green and red from attractors corresponding to 3°, 2° and 1° fates, respectively. Dark green region corresponds to the set $C_2 = \{(x,y) \in \mathbb{R}^2 : \|(x,y) - (x_2^*, 0)\| \leq R\|(x_s^*, 0) - (x_2^*, 0)\|$. Red green region corresponds to the set $C_1 = \{(x,y) \in \mathbb{R}^2 : \|(x,y) - (x_1^*, 0)\| \leq Smin\{\|(x_s^*, 0) - (x_1^*, 0)\|, M - \sqrt{-c_{PC}}\}$

the parameter vector that represents WT conditions in the model, we can simulate all the mutants in Table 1 in the main text by changing the corresponding parameters the following way.

***let-23* mosaic** In this mutant worm, there are no EGF receptors in P5.p. Therefore we simulate this mutant with the same parameter vector Θ_{WT} as the WT but we change the parameter that controls the EGF signal for P5.p. In particular, we set $s = 0$ only in P5.p for this mutant.

***let-23* mosaic** In this mutant worm, there are no EGF receptors in P5.p. Therefore we simulate this mutant with the same parameter vector Θ_{WT} as the WT but we change the parameter that controls the EGF signal for P5.p. In particular, we set $s = 0$ only in P5.p for this mutant.

Half dose of *lin-3* In this case, the EGF ligand is reduced by half with respect to the WT case in all the VPCs. We can translate this into our model by taking the parameter $s/2$ instead of s in the WT parameter vector.

Half dose of *lin-12* In this case, the Notch receptor is reduced by half with respect to the WT case in all the VPCs. To do this we reduce by half the WT parameter l.

Notch null, 2× WT EGF In this mutant, the VPCs lack the Notch receptor, and the EGF that they receive is twice as much the EGF signal that they receive in the WT worm. Taking this into account, we simulate this mutant by doubling s and setting l to zero in the WT parameter vector.

No notch signalling, WT EGF As in the previous case, the VPCs do not receive any Notch signal because they lack the Notch receptor. However, in this case, EGF signal is the same as in the WT case. We can translate this into our model by setting l to zero in the WT parameter vector.

Excess EGF These mutants are modelled as a multiplicative increase in EGF signal. For the mutant JU1100, the VPCs receive a higher EGF signal but the fold change is unknown. We add a parameter $s_{eE} > 0$ to the model, that will represent the fold change of EGF mutant in this mutant with respect to the WT case. Therefore we multiply s by s_{eE} in the WT parameter vector. On the other hand, in the case of JU2113 it is known that the EGF level is increased by 1.25-fold, so for this mutant we multiply s by 1.25.

Reduced Notch These mutants are modelled as a multiplicative decrease in Notch signal. In particular, we consider a reduction of $0.4 \times l$, as explained in the main text.

Excess EGF & ectopic Notch Excess EGF is modelled as explained above, while ectopic Notch is modelled as an additive increase in the signal, where now the θ_{N} described in Eqs. 41,42, and 43 become $\theta_{\mathrm{N}}^i = \theta_{\mathrm{N}}^{i,WT} + l\theta_{\mathrm{N}}^0$, where θ_{N}^0 is constant and equal to 0.12.

Reduced EGF Reduced EGF is modelled as a multiplicative decrease in EGF signal. For the mutants considered, the VPCs receive a low EGF signal but the fold change is unknown. We consider a parameter $1 > s_{rE} > 0$ to the model, that will represent the fold change of EGF mutant in this mutant with respect to the WT case. Therefore we multiply s by s_{rE} in the WT parameter vector. In our case, $s_{rE} = 0.36$.

AC ablation mutants These mutants correspond to experiments in which the AC is ablated (i.e. EGF signal is removed) at different stages of the development of the worm. If we simulate the competence time of the WT case from $t = 0$ to $t = 1$, we simulate the competence time of these mutants by taking the parameter vectors:

$$\begin{cases} \Theta_{WT} & t \in [0, t_{ACAb}] \\ \Theta_{ACAb} & t \in (t_{ACAb}, 1] \end{cases}$$

where Θ_{ACab} is obtained by setting $s = s\frac{(1+\tanh(H_E t_{ACAb}+M_E))}{2}e^{-\lambda_E(t-t_{ACAb})}$ in the WT parameter vector, meaning that EGF is removed so it decays exponentially from the point to which it reached at t_{ACAb}, and t_{ACAb} represents the developmental stage. Since the numerical timing of each developmental stage is not available, we first followed the approach taken by [5, 6] and assumed that the AC ablation times were uniformly distributed in $[0.2, 0.8]$, as shown in Table SI3. However, after fitting the data corresponding to the stages L2 Lethargus and DU divided, the data suggested a different correspondence, as shown in Table Table SI3. This change is equivalent to a change in the EGF monotone function, $\sigma(\Psi(t))$, that we assumed to be sigmoidal (see Fig. SI17) and is, in fact, is consistent with data in [13], where the expression of an EGF pathway transcriptional reporter *egl-17::CFP* is measured at some developmental stages. It could also be achieved by locally changing the flow around the saddle. However, we find the change in the EGF function is not very significant, and due to the lack of data for EGF dynamics, we accept this change.

**Developmental stage**	**Initial t_{ACAb}**	**$\Psi(t_{ACAb})$ from fitting**
L2 lethargus	0.2	0.2
Early L3	0.32	0.34
DU divided	0.44	0.40
VU divided	0.56	0.45
3° divided	0.68	0.49
2-cell stage	0.8	0.78

Table SI3: Table of correspondance between developmental stages and modelling time.

3 Parameter estimation

We take advantage of sequential Monte Carlo ABC (ABC SMC) algorithm in [14]. This version of the ABC algorithm borrows ideas from importance sampling and sequential Monte Carlo, as the name suggests. The ABC SMC was first proposed by [15], and later corrected by [16, 17, 14]. Various ABC SMC algorithms are proposed in the literature (see for example [16, 18, 15, 14]), but here we focus on the fairly general ABC SMC algorithm by [14]. The ABC SMC sampler methodology approximates a sequence of probability distributions $\{\pi_t\}_{0\leq t\leq T}$ that satisfies the condition that $d(\boldsymbol{X}(\boldsymbol{\theta})), \boldsymbol{X}_0) \leq \varepsilon_t$,

$$\{\pi_t\}_{0\leq t\leq T} = \{\pi(\boldsymbol{\theta} \mid d(\boldsymbol{X}(\boldsymbol{\theta})), \boldsymbol{X}_0) \leq \varepsilon_t)\}_{0\leq t\leq T}. \tag{47}$$

In order to produce these probability distributions, the algorithm starts by sampling parameter values $\boldsymbol{\theta}^*$ from a prior distribution $\pi(\boldsymbol{\theta})$. Then, the algorithm accepts N parameter values that satisfy $d(\boldsymbol{X}(\boldsymbol{\theta}^*)), \boldsymbol{X}_0) \leq \varepsilon_1$. It assigns a set of equal weights, $\{\omega_1^{(i)} = 1/N\}_{i=1}^N$, to each accepted parameter value $\boldsymbol{\theta}_1^{(i)}$, $i = 1, \ldots, N$, in this context called particles.

Then the algorithm proceeds in a sequential manner. In each step t, a set of N particles $\{\boldsymbol{\theta}_t^{(i)}\}_{i=1}^N$ is generated. This is done by first sampling a particle $\boldsymbol{\theta}^{**}$ from the discrete distribution with support the

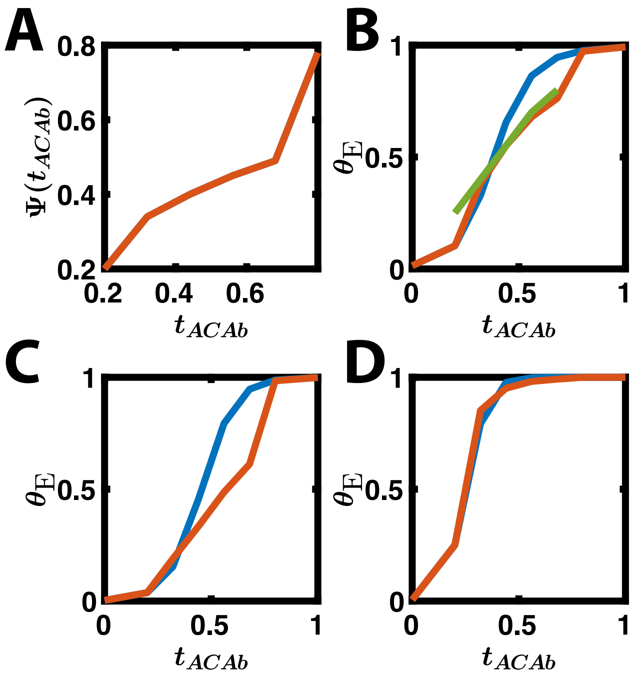

Figure SI17: (A) Change in time Ψ obtained from fitting. (B) In blue, the mean over all the particles of the original EGF monotone sigmoidal function, $\sigma(t)$. In orange, the mean over all the particles of the modified EGF monotone function, $\sigma(\Psi(t))$. In green, scaled *egl-17* expression measured in [13]. (C-D) In blue, the original EGF monotone sigmoidal function, $\sigma(t)$, in orange, the modified EGF monotone function, $\sigma(\Psi(t))$, for different parameter values

finite set of particles generated in the previous step, $\{\boldsymbol{\theta}_{t-1}^{(i)}\}_{i=1}^{N}$, and probabilities given by the weights $\{\omega_{t-1}^{(i)}\}_{i=1}^{N}$. Then, the sample is perturbed using the Markov kernel function $K_t(\boldsymbol{\theta}^*, \boldsymbol{\theta}^{**})$. These steps are repeated until N particles $\boldsymbol{\theta}^*$ that satisfy the conditions $\pi(\boldsymbol{\theta}^*) > 0$ and $d(\boldsymbol{X}(\boldsymbol{\theta}^*), \boldsymbol{X}_0) \leq \varepsilon_t$ are found.

Algorithm 1: ABC SMC algorithm

```
Initialize ε_1, ..., ε_T
for i = 1, 2, ..., N do
    repeat
        Generate θ* from the prior distribution π(·)
    until d(X(θ*), X_0) ≤ ε_t;
    Set θ_1^(i) = θ* and set the weight ω_1^(i) for particle θ_1^(i) equal to 1/N.
for t = 2, ..., T do
    for i = 1, 2, ..., N do
        repeat
            Generate θ** from the previous population {θ_{t-1}^(i)}_{i=1}^N
            Perturb the particle to obtain θ* ~ K_t(·, θ**), where K_t is a perturbation kernel.
        until π(θ*) > 0 and d(X(θ*), X_0) ≤ ε_t;
        Set θ_t^(i) = θ* and calculate the weight for particle θ_t^(i), ω_t^(i) = π(θ_t^i) / Σ_{j=1}^N ω_{t-1}^(j) K_t(θ_{t-1}^(j), θ_t^(i))
    Normalise the weights.
```

For this implementation, we first need to study the dependence on the parameters of our model. We will study the range of the parameters, consider some constraints that the model and certain mutants in Table 1 impose on the parameters, and provide the corresponding priors.

Secondly, we need to define a distance function d that will measure the similarity between the

experimental data set and the corresponding simulated data set, i.e. the goodness of fit.

For the implementation of the ABC SMC method, we also need to define a decreasing sequence of thresholds $\{\varepsilon_t\}_{t\geq 0}$ that will determine the maximum distance between the experimental data and the simulated data in each step t of the algorithm. These, in turn, define the intermediate distribution p_{ε_t} from which the algorithm samples at step t.

Also, we need to choose the number N of particles sampled from the distribution p_{ε_t} at each step t.

Finally, we need to provide the perturbation kernels $\{K_t(\cdot \mid \cdot)\}_{t=1}^{N}$ that will set the limits of exploration of the parameter space in each step.

We will discuss these points further in the following subsections. These steps are implemented in Matlab, and the code is provided in the following github repository:

https://github.com/ecamacho90/VulvalDevelopment

3.1 Constraints on the parameters

In Table SI1, we summarised the constraints that the transformation from the signal space to the control space imposes on the parameters. In the following subsections we will outline three other constraints imposed on the parameters by the model and the data in Table 1 in the main text.

3.1.1 Constraint imposed by the sigmoidal function $\chi(y)$

As mentioned above, we approximate the indicator function $H(y)$ by a sigmoidal function that has the form

$$\chi(y) = \frac{1 + \tanh\left(H\left(-y + rM\right)\right)}{2} \tag{48}$$

where we choose $r = \frac{\sqrt{2}-1}{2\sqrt{2}}$.

Focusing on the deterministic system at first, the trajectory corresponding to the state of a VPC is moving on a flow characterised by the equilibria of the system:

$$\begin{cases} \frac{dx}{dt} &= \frac{1}{\tau}[\chi(y)(-4x^3 - 2ax - b) - (1 - \chi(y))x] \\ \frac{dy}{dt} &= -\frac{1}{\tau}[y((y - M)^2 + c)]. \end{cases} \tag{49}$$

This is similar to Eq. 2 in the main text, but we have substituted the Heavyside function by the sigmoidal function. The control parameters a, b, c are functions of the signals as we explained before.

As studied above, the critical points of the system in Eq. 49 will have y-coordinates equal to 0, $M - \sqrt{-c}$ and $M + \sqrt{-c}$ (these two latter ones only when $c < 0$).

The constraint that we introduce here is firstly due to the fact that $\chi(0)$ needs to be approximately equal to 1, so that the values of the x-coordinates of the critical points come only from the cusp equation. Secondly, $\chi(M - \sqrt{-c})$ and $\chi(M + \sqrt{-c})$ need to be approximately equal to zero so that there is only one possible value of the x-coordinate for these critical points and it must be equal to zero.

Consequently, we need to impose:

1. $\chi(0) \approx 1$

   This condition can be translated into the condition $1 \geq \chi(0) \geq 1 - \varepsilon$ where ε is small.

   Substituting in Eq. 48 we get:

   $$\chi(0) = \frac{1 + \tanh\left(H\left(rM\right)\right)}{2} \geq 1 - \varepsilon \Leftrightarrow M \geq \frac{\operatorname{atanh}(1 - 2\varepsilon)}{rH}$$

   This constrains the smallest value that the parameter $M > 0$ can take. This minimum value of M will depend on how accurate we would like the function $\chi(y)$ to be or, in other words, how small ε is, as well as on the steepness H of the sigmoidal function. We choose $\varepsilon = 10^{-12}/2$, and leave $H > 0$ as a parameter to be estimated. Therefore

   $$M \geq M_{min}(H) = \frac{\operatorname{atanh}(1 - 10^{-12})}{\frac{\sqrt{2}-1}{2\sqrt{2}} H}. \tag{50}$$

See Fig. SI18 for a graphical explanation.

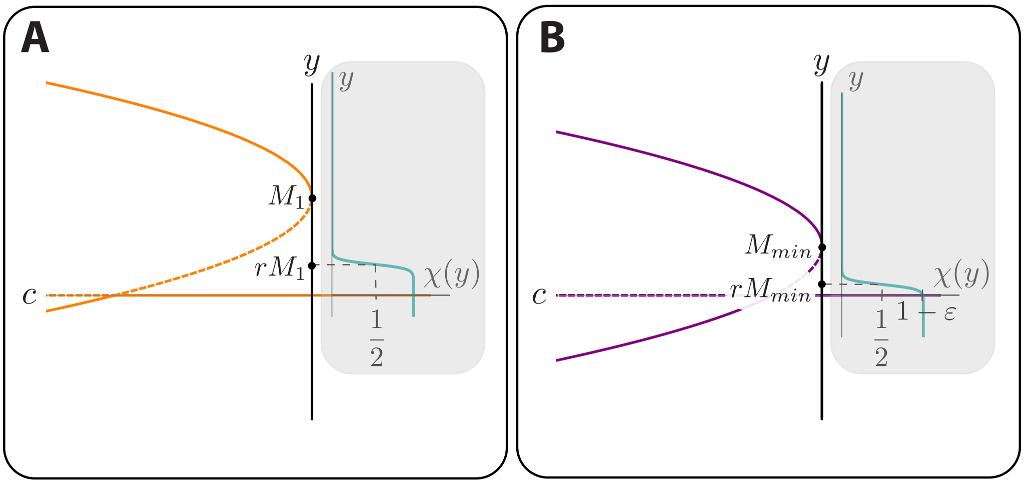

Figure SI18: Bifurcation diagram of the y-coordinate with respect to the control parameter c, together with a plot of $\chi(y)$, for $M = M_1$ (on the left plot (A)) and $M = M_{min}$ (on the right plot (B)). Fixed a value of H, the value of M "*shifts*" the graph of $\chi(y)$ along the y-axis, by changing the value of rM where $\chi(rM) = 1/2$. The minimum value M_{min} that M can take is such that $\chi(0) = 1-\varepsilon$, otherwise $\chi(0)$ would be "too far" from 1.

2. $\chi(y) \approx 0$ when $y > M - \sqrt{-c}$ for all M and c allowed.

   This constraint is necessary so that the value of $\chi(y)$ evaluated at the critical points $y = M - \sqrt{-c}$, $y = M + \sqrt{-c}$ is approximately zero for all values of M and c allowed.

   We know that the sigmoidal function is monotonically decreasing, therefore $\chi(M + \sqrt{-c}) \leq \chi(M - \sqrt{-c})$. This means that we only need to impose $\chi(M - \sqrt{-c}) \leq \varepsilon$, where ε takes the same value as before ($\varepsilon = 10^{-12}/2$).

   To achieve that, we restrict the value of c to be bigger than a value c_{min}, where c_{min} is such that $\chi(M - \sqrt{-c_{min}}) = \varepsilon$. If $c < c_{min}$ then $\chi(M - \sqrt{-c}) > \varepsilon$, because $\chi(y)$ is monotonically decreasing. Therefore we should avoid values $c < c_{min}$ (see Fig. SI19).

   We should therefore impose:

$$c > c_{min} = \min_c\{\chi(M - \sqrt{-c}) \leq \varepsilon\} \tag{51}$$

   Now,

$$\chi(M - \sqrt{-c_{min}}) = \frac{1 + \tanh\left(H\left(-M + \sqrt{-c_{min}} + rM\right)\right)}{2} = \varepsilon$$

   implies that

$$c > c_{min} = -\left(\frac{\operatorname{atanh}(1-2\varepsilon)}{H} + rM - M\right)^2. \tag{52}$$

   But in our model, the control parameter c is written as a function of the signals. In particular:

$$c(\text{EGF,NOTCH}) = m_{31}EGF + m_{32}NOTCH + q_3.$$

   Considering the forms of the θ_{E} and functions (see Eq. 40, 41, 42, 43, 44) and the fact that $m_{31}, m_{32} > 0$ (see Table SI1) we know that $c(\text{EGF,NOTCH}) \geq q_3$.

   This leads us to the final condition:

$$q_3 > -\left(\frac{\operatorname{atanh}(1-2\varepsilon)}{H} + rM - M\right)^2, \tag{53}$$

   where $r = (\sqrt{2}-1)/2\sqrt{2}$ and $\varepsilon = 10^{-12}/2$.

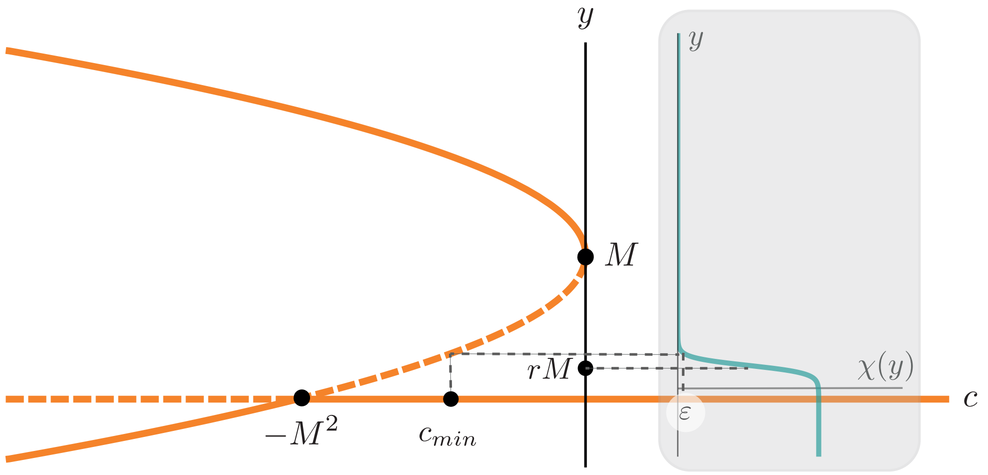

Figure SI19: Bifurcation diagram of the y coordinate together with a plot of $\chi(y)$. $\chi(y) = 1/2$ when $y = rM$. The value c_{min} is such that $\chi(M - \sqrt{c_{min}}) = \varepsilon$.

3.1.2 Constraint imposed by mutant (5) Notch null, 2xWT EGF

For this mutant, there is no Notch signal in any of the VPCs since they lack the Notch receptor. This means that, in our model, the θ_{N} coordinate is zero for all the cells at all times, i.e. $l = 0$.

In Table 1 we can see that P5.p and P6.p adopt fate 1°. However, in our model, P5.p and P6.p could only adopt fate 1° if their trajectory escapes the basin of attraction of the attractor representing fate 3° when the signals are not present. This will only happen if the attractor corresponding to 3° fate bifurcates away for the values of the control parameters in Eq. 39, where $\theta_{\text{N}} = 0$ and θ_{E} is multiplied by two with respect to WT.

Since we know that θ_{E} increases in time for both cells, and that their maximum value is equal to $\theta_{E2} = 2s\gamma$ for P5.p and $\theta_{E3} = 2s$ for P6.p, this gives us the equations

$$\begin{aligned} c_3 &= 2s + q_3 \geq 0 \\ c_2 &= 2s\gamma + q_3 \geq 0. \end{aligned} \tag{54}$$

However, since $\gamma \in (0, 1)$ and $s > 0$, we only need to impose $2s\gamma + q_3 \geq 0$.

3.1.3 Constraint imposed by mutant (6) Notch null, WT EGF

As before, there is no Notch signal in any of the cells and EGF is constant and equal to the WT signal. This means that, in our model, the θ_{N} coordinate is zero for all the cells at all times, i.e. $\theta_{\text{N}} = 0$. As before, P6.p assumes primary fate (see Table 1 in the main text). With a similar reasoning as the one given before, this can only happen if the control parameters corresponding to P6.p satisfy the condition $c_3 = s + q_3 \geq 0$.

3.2 Ranges and priors

The range of values that each parameter can take is given on Table SI4. These ranges are imposed by the model definition and constraints in Table SI1.

In order to define the priors, we need to take into account their ranges and the constraints imposed on them by the data and by the model. The priors are chosen so that they are fairly non informative but still reflecting our knowledge about their ranges.

For bounded parameters $m_{11}, m_{12}, m_{21}, \gamma$, $n_{1,y}$ and l_d we assign uniform priors on their range.

Positive non-bounded parameters $1/\tau, H, H_1, H_2, \|n_1\|, s, l$ and s_E are assigned Gamma priors $\text{Ga}(\kappa, \beta)$ where κ and β are the shape and scale of the distribution, respectively. Note that the probability density function of $U \sim \text{Ga}(\kappa, \beta)$ is given by

$$\pi_U(u) = \frac{\beta^\kappa}{\Gamma(\kappa)} u^{\kappa-1} e^{-\beta u}, \tag{55}$$

Parameters	Range	Prior
$1/\tau$	$(0,\infty)$	$\mathrm{Ga}(2,2)$
M	$(0,\infty)$	$M_{min} + \mathrm{Ga}(2,2)$
m_{11}, m_{12}	$[0,1]$	$\mathcal{U}([0,1])$
m_{21}	$[-1,1]$	$\mathcal{U}([-1,1])$
q_1, q_2	$\mathbb{R}$	$\mathcal{N}(0,2)$
q_3	$(-\infty,0)$	$\mathrm{Ga}(2,2)$
γ	$[0,1]$	$\mathcal{U}([0,1])$
n_0	$\mathbb{R}$	$\mathcal{N}(0,1)$
$\lvert\lvert n_1\rvert\rvert$	$(0,\infty)$	$\mathrm{Ga}(2,2)$
$n_{1,y}$	$[-1,1]$	$\mathcal{U}([-1,1])$

Parameters	Range	Prior
s, l	$(0,\infty)$	$\mathrm{Ga}(2,2)$
α	$(0,\infty)$	$\mathrm{Exp}(1)$
l_d	$[0,1]$	$\mathcal{U}([0,1])$
λ_E	$(0,\infty)$	$\mathrm{Ga}(3/2,4)$
λ_N	$(0,\infty)$	$\mathrm{Ga}(3/2,4)$
H_E	$(0,\infty)$	$\mathrm{Ga}(3/2,4)$
M_E	$\mathbb{R}$	$\mathcal{N}(0,2)$
s_E	$(0,\infty)$	$\mathrm{Ga}(2,2)$
σ_{dif}	$(0,\infty)$	$\mathcal{U}([0.05,1])$

Table SI4: Tables of ranges and priors of the parameters.

where Γ is the gamma function. We choose $\kappa = 2$, $\beta = 2$, and in this case $\Gamma(\kappa) = \Gamma(2) = (2-1)! = 1$, or $\kappa = 3/2$, $\beta = 4$ for parameters where we would like to favor smaller values. For the positive and non-bounded parameter M, since it needs to be bigger than $M_{min}(H)$ (see Eq. 50), we impose $M \sim M_{min}(H) + \mathrm{Ga}(2,2)$.

On the other hand, M_1, M_2, q_1, n_0 and q_2 are assigned Normal priors $\mathcal{N}(\mu, \sigma)$ where μ is the mean of the distribution and σ is the standard deviation. Note that the probability density function of $U \sim \mathcal{N}(\mu, \sigma)$ is given by

$$\pi_U(u) = \frac{1}{\sqrt{2\pi\sigma^2}} e^{-\frac{(u-\mu)^2}{2\sigma^2}}. \tag{56}$$

We choose $\mu = 0$ and $\sigma = 1$ or 2.

For the parameter α we would like to favour smaller values, so we choose an exponential prior $\mathrm{Exp}(\lambda)$, where the probability density function of $U \sim \mathrm{Exp}(\lambda)$ is

$$\pi_U(u) = \frac{1}{\lambda} e^{-\frac{u}{\lambda}}. \tag{57}$$

We choose $\lambda = 1$.

The parameter q_3 is a negative non-bounded parameter, so we impose $q_3 \sim -\mathrm{Ga}(2,2)$.

And finally, since we would like to keep the noise level relatively low, we give σ_{dif} a flat prior on $[0.05, 1]$, i.e. a uniform distribution $\mathcal{U}([0.05,1])$.

Table SI4 contains a list of the priors for each parameter.

3.3 Distance

In order to approximate the posterior distribution of the parameters, the ABC SMC algorithm computes a sequence of intermediate distributions that are obtained by comparing the simulated data and the experimental data. This comparison is made by means of a distance function d that measures the level of similarity between two data sets.

As explained in the main text, Table 1 provides 171 data points or probabilities which correspond to the proportion of times that VPC c ($c = 1,2,3$ for P4.p, P5.p and P6.p respectively), adopted fate f ($f = 1,2,3$ for $1°, 2°$ and $3°$ respectively) in experiment e ($e = 1, 2, \ldots, 21$ for the corresponding experiment in Table 1). Each data point will be represented by $p_{e,f,c}$.

Given a parameter vector $\boldsymbol{\theta}$, we can simulate each data point using our model to obtain the corresponding $p^{sim}_{e,f,c}(\boldsymbol{\theta})$.

If $\boldsymbol{X}_0^{TD}$ is the subset of data corresponding to experiments in the training data set (see Table 1), we define the distance between $\boldsymbol{X}_0^{TD}$ and the corresponding simulation $\boldsymbol{X}^{TD}(\boldsymbol{\theta})$ as

$$d(\boldsymbol{X}_0^{TD}, \boldsymbol{X}^{TD}(\boldsymbol{\theta})) = \frac{1}{E} \sum_{e \in TD} \sum_{f=1}^{3} \sum_{c=1}^{3} \left| p_{e,f,c} - p^{sim}_{e,f,c}(\boldsymbol{\theta}) \right| + \frac{1}{E} \sum_{e \in TD} \sum_{c=1}^{3} \left| p^{sim}_{e,4,c}(\boldsymbol{\theta}) \right|, \tag{58}$$

where E is the number of experiments in the training data set, $p_{e,f,c}$ and $p^{sim}_{e,f,c}(\boldsymbol{\theta})$ are the experimental and simulated probabilities of cell c becoming fate f in experimental condition e, respectively, and $p^{sim}_{e,4,c}$ is the proportion of times that our model could not assign a fate to cell c when simulating experiment e.

3.4 Sequence of thresholds and number of particles

The ABC SMC algorithm approximates the posterior distribution of the parameters by sequentially sampling from the distributions in Eq. 47. For this, one needs to choose a decreasing sequence of tolerances $\varepsilon_1 > \varepsilon_2 > \ldots \varepsilon_T$.

The most common approach is to choose ε_{t+1} to be the α quantile of the population of particles obtained at time t [16, 19], taking into account a trade off between acceptance rate of the particles and the successful convergence of the method [20]. Here we use $\alpha = 0.3$ quantile, so the method determines the value of the threshold ε_t at the beginning of the step t of the algorithm by sorting the distances obtained at the previous step and setting ε_t such that the 0.3% of the simulated data at step $t-1$ are below it. In our case, this sequence is given by $\varepsilon_1 = 5 > \ldots > 0.43 = \varepsilon_{14}$. We decided to stop at this threshold value because the resemblance between the simulated data and the training data was good and the variation across particles suggested that reducing the value further would result in overfitting.

An advantage of ABC SMC is that it can be easily parallelised since the N particles corresponding to a step t are sampled independently. With that in mind, taking into account the number of parameters to be fitted and the computational time of the simulations, we choose to sample $N = 2 \times 10^4$ particles at each step t of the algorithm, and parallelise the computations, streaming computation into 100 jobs which compute 500 subsets of 40 particles each, significantly reducing the computation time of the algorithm.

3.5 Perturbation kernel

The choice of perturbation kernel is also very important as it can speed up the convergence of the method [21] if it samples from the regions of interest of parameter space.

For simplicity, let us denote by $\boldsymbol{X}_0^{TD}$ the set of training data. We denote the i-th particle obtained at step $t-1$ as $\boldsymbol{\theta}^{(i,t-1)}$, its corresponding weight as $w^{(i,t-1)}$ and the data simulated with $\boldsymbol{\theta}^{(i,t-1)}$, for the same experiments as in $\boldsymbol{X}_0^{TD}$, as $\boldsymbol{X}(\boldsymbol{\theta}^{(i,t-1)})$. It holds that

$$d(\boldsymbol{X}_0, \boldsymbol{X}(\boldsymbol{\theta}^{(i,t-1)})) \leq \varepsilon_{t-1} \quad \forall 1 \leq i \leq N, \tag{59}$$

where N is the number of sampled particles at each step.

The particles at step t are computed by randomly choosing a particle $\boldsymbol{\theta}^{(*,t-1)}$ from the previous population $\{\boldsymbol{\theta}^{(i,t-1)}\}_{i=1}^N$ with weights $\{\omega^{(i,t-1)}\}_{i=1}^N$, perturbing it with a chosen kernel $K_t(\cdot|\boldsymbol{\theta}^{(*,t-1)})$ and checking that the candidate parameter vector $\boldsymbol{\theta}^{**}$ obtained from that perturbation satisfies $d(\boldsymbol{X}_0, \boldsymbol{X}(\boldsymbol{\theta}^{**})) < \varepsilon_t$.

Many types of perturbation kernels $K_t(\cdot|\cdot)$ can be used. The most simple choice would be the component-wise perturbation kernel, in which the parameter vector $\boldsymbol{\theta} = (\theta_1, \ldots, \theta_m)$ (m being the number of parameters to be estimated) is perturbed component-wise. In other words, each parameter θ_j is perturbed according to a Gaussian distribution $K_t(\cdot|\theta_j) = K(\cdot|\theta_j) = \mathcal{N}(\theta_j, \sigma_j)$ or Uniform distribution $\mathcal{U}([\theta_j - \sigma_j, \theta_j + \sigma_j])$. Note that, in this case, there is a kernel distribution for each component of the parameter vector and it is independent of the other components. Moreover, the kernel distributions are the same for all t. As a result, these kernels are not very efficient since they do not take into account the fact that some parameters can have a certain correlation.

Following the study in [21] we decide to implement the multivariate normal kernel with optimal covariance matrix (OLCM). This perturbation kernel considers a multivariate normal distribution around each particle (assessing the correlation between the parameters) and, moreover, the covariance matrix will differ from particle to particle, taking into account the structure of the *good* particles sampled. Let us define the following set

$$\left\{\left(\tilde{\boldsymbol{\theta}}^{(k,t-1)}, \tilde{\omega}^{(k,t-1)}\right)\right\}_{k=1}^{N_0} = \left\{\left(\boldsymbol{\theta}^{(i,t-1)}, \frac{\omega^{(i,t-1)}}{\bar{\omega}}\right) : d(\boldsymbol{X}_0, \boldsymbol{X}(\boldsymbol{\theta}^{(i,t-1)})) \leq \varepsilon_t, 1 \leq i \leq N\right\}, \tag{60}$$

where $\bar{\omega}$ is a normalising constant such that $\sum_{k=1}^{N_0} \tilde{\omega}^{(k,t-1)} = 1$. The set in Eq. 60 is the set of particles of the population obtained at time $t-1$ for which the simulated data is closer to the experimental data than the current threshold ε_t, i.e. the set of *good* particles at time $t-1$. The weights $\bar{\omega}$ are obtained by normalising this subset of N_0 particles.

In this approach, the perturbation kernel follows a normal distribution, $K_t(\cdot \mid \boldsymbol{\theta}^{(*,t-1)}) \sim \mathcal{N}(\boldsymbol{\theta}^{(*,t-1)}, \boldsymbol{\Sigma}^{(t)}_{\boldsymbol{\theta}^{(*,t-1)}})$, which is centered at the particle that will be perturbed $\boldsymbol{\theta}^{(*,t-1)}$ with covariance matrix also dependent on the particle value. [21] propose the optimal covariance matrix $\boldsymbol{\Sigma}^{(t)}_{\boldsymbol{\theta}^{(*,t-1)}}$ to be equal to the covariance of the set of particles from step $t-1$ whose distance is smaller than the current threshold ε_t (i.e. particles in the set described in Eq. 60) plus a bias term related to the discrepancy between the mean of the particles in such population and the particle of interest $\boldsymbol{\theta}^{(*,t-1)}$:

$$\boldsymbol{\Sigma}^{(t)}_{\boldsymbol{\theta}^{(*,t-1)}} \approx \sum_{k=1}^{N_0} \tilde{\omega}^{(k,t-1)} \left(\tilde{\boldsymbol{\theta}}^{(k,t-1)} - \tilde{\boldsymbol{m}}\right) \left(\tilde{\boldsymbol{\theta}}^{(k,t-1)} - \tilde{\boldsymbol{m}}\right)^T + \left(\tilde{\boldsymbol{m}} - \boldsymbol{\theta}^{(*,t-1)}\right) \left(\tilde{\boldsymbol{m}} - \boldsymbol{\theta}^{(*,t-1)}\right)^T, \quad (61)$$

where $\tilde{\boldsymbol{m}} = \sum_{k=1}^{N_0} \tilde{\omega}^{(k,t-1)} \tilde{\boldsymbol{\theta}}^{(k,t-1)}$ is the mean of the population of particles described in Eq. 60. We therefore implement these multivariate normal perturbation kernels $K_t(\cdot \mid \boldsymbol{\theta}^{(*,t-1)}) \sim \mathcal{N}(\boldsymbol{\theta}^{(*,t-1)}, \boldsymbol{\Sigma}^{(t)}_{\boldsymbol{\theta}^{(*,t-1)}})$ with optimal covariance matrix given in Eq. 61.

4 Fitting results

4.1 Software run-time

It took a mean of 3.5 seconds to simulate all the mutants in the Training Data set given one particle that satisfied all the constraints and priors. The time to run the ABC algorithm depended on the step of the algorithm and number of particles to be found. For N=20,000 particles, the time increased as the algorithm is challenged with smaller thresholds, ranging from a mean of 3.5 seconds/particle on the first step of the algorithm to a mean of 56.15 seconds/particle on the last step of the algorithm. This means, to compute 20,000 particles with 4 cores or parallel workers it ranged from 4.8 hours on the first step to 78 hours on the last one..

4.2 Approximated posteriors

In S1 Fig. we show the evolution of the approximate posteriors of the parameters from the first step of the algorithm until the last step of the algorithm, which is quantified in Table SI5.

**Parameter**	**Prior**	**App. Post.**
$1/\tau$	4.02 ± 2.84	5.23 ± 2.47
M	7.87 ± 2.83	6.58 ± 1.53
m_{11}	0.5 ± 0.29	0.26 ± 0.17
m_{12}	0.5 ± 0.29	0.38 ± 0.2
m_{21}	0 ± 0.58	0.64 ± 0.1
q_1	0 ± 2	-2.17 ± 0.7
q_2	0.02 ± 2	-0.37 ± 0.6
q_3	-4.01 ± 2.83	-1.45 ± 0.64
γ	0.5 ± 0.29	0.22 ± 0.06
n_0	0 ± 1	-0.62 ± 0.71
$\lvert\lvert n_1 \rvert\rvert$	4 ± 2.86	4.41 ± 2.2

**Parameter**	**Prior**	**App. Post.**
$n_{1,y}$	0 ± 0.58	-0.49 ± 0.24
s	4.03 ± 2.86	7.6 ± 2.73
l	3.98 ± 2.81	6.26 ± 2.61
α	1 ± 1	0.5 ± 0.43
l_d	0.5 ± 0.29	0.48 ± 0.27
λ_E	5.99 ± 4.89	8.72 ± 4.26
λ_N	6.04 ± 4.95	9.7 ± 7.37
H_E	5.97 ± 4.89	5.95 ± 2.62
M_E	0 ± 1.99	-2.23 ± 0.85
s_E	3.99 ± 2.8	4.06 ± 2.12
σ_{dif}	0.53 ± 0.27	0.23 ± 0.16

Table SI5: Tables of priors and approximated posteriors of the parameters at the last step of the algorithm (means and standard deviations.)

4.3 Exploring the single-cell fate map

We also studied how the data constrained the single-cell fate map in the signal space, given by the map between the control space and the biological signals.

As we described before, the non-vulval to vulval transition is determined by the line $r_c = \{(\theta_{\mathrm{E}}, \theta_{\mathrm{N}}) : \theta_{\mathrm{N}} = \tan(\omega_c)\theta_{\mathrm{E}} - q_3/m_{32}l\}$ where $\omega_c = -m_{31}s/m_{32}l$, that is, when the control parameter c is equal

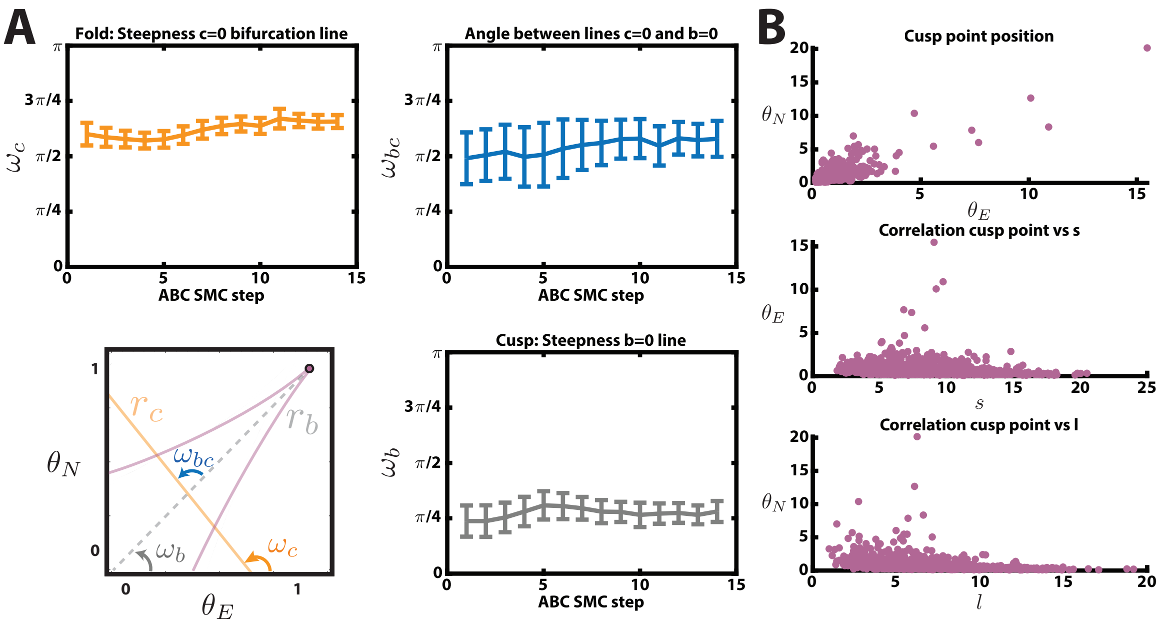

Figure SI20: Data-fitted single-cell fate map. (A) Evolution of the steepness of the fold line r_c (orange), the steepness of the cusp mid-line r_b (gray) and the angle between them (blue) over the steps of the ABC algorithm. (B) Distribution of the positions of the cusp point for each particle obtained in the last step of the algorithm. Top: Coordinates of such cusp points in the $(\theta_\mathrm{E}, \theta_\mathrm{N})$ space. Middle: Correlation between EGF scaling parameter s and position of the cusp point on the EGF-axis. Bottom: Correlation between Notch scaling parameter l and position of the cusp point on the NOTCH-axis.

to zero; while the primary to secondary fate transition is determined by the cusp, which mid-line is determined by the line $r_b = \{(\theta_\mathrm{E}, \theta_\mathrm{N}) : \theta_\mathrm{N} = \tan(\omega_b)\theta_\mathrm{E} - q_2/m_{22}l\}$ where $\omega_b = -m_{21}s/m_{22}l$, that is, when the control parameter b is equal to zero (see Eq. 39) (see Fig. SI20A). It is important to note that θ_E and θ_N have arbitrary units. And therefore, understanding the positions of r_c and r_b can give us information about how signal perturbations can affect the differentiation of a single cell and, in turn, vulval patterning.

Interestingly, even though we did not impose any constraints on the steepness of the lines r_c and r_b, the results of fitting suggest that they are strongly constrained by the data (Fig. SI20A). We find that the angle that forms the line r_c with the θ_E axis, ω_c, is always between $\pi/2$ and $3\pi/4$, converging to a mean steepness of -2.16 ± 0.5 after the 14 steps of ABC SMC (Fig. SI20).

Similarly, we observe that the steepness of the r_b line is also strongly constrained by the data, converging to a positive value of 1.66 ± 0.65 after the 14 steps of ABC SMC (Fig. SI20A). Moreover, we observe that the two lines r_c and r_b normally intersect in an angle of close to $\pi/2$.

The position of the cusp point within the r_b line, however, is more variable, as shown in Fig. SI20B. We wondered whether this variability is due to the degree of freedom given by the choice of the scaling parameters s and l. However, we see that there is no correlation between these two parameters and the position of the cusp point (Fig. SI20B).

References

[1] Tim Poston and Ian Stewart. *Catastrophe theory and its applications.* Courier Corporation, 2014.

[2] John Guckenheimer. The catastrophe controversy. *The Mathematical Intelligencer*, 1(1):15–20, mar 1978.

[3] P W Sternberg. Vulval development, *WormBook*, ed. *The C. elegans Research Community, WormBook*, 2005.

[4] Paul W. Sternberg and H.Robert Horvitz. The combined action of two intercellular signaling pathways specifies three cell fates during vulval induction in *C. elegans*. *Cell*, 58(4):679–693, 1989.

[5] F Corson and E D Siggia. Geometry, epistasis, and developmental patterning. *Proceedings of the National Academy of Sciences*, 109(15):5568–5575, 2012.

[6] Francis Corson and Eric D Siggia. Gene free methodology for cell fate dynamics during development. *eLife*, 6:e30743, 2017.

[7] Daniel D. Shaye and Iva Greenwald. Endocytosis-mediated downregulation of LIN-12/Notch upon Ras activation in *Caenorhabditis elegans*. *Nature*, 420(6916):686–690, dec 2002.

[8] Paul Fearnhead, Vasilieos Giagos, and Chris Sherlock. Inference for reaction networks using the linear noise approximation. *Biometrics*, 70(2):457–466, 2014.

[9] Edward WJ Wallace. A simplified derivation of the linear noise approximation. *arXiv preprint arXiv:1004.4280*, 2010.

[10] Desmond J. Higham. An algorithmic introduction to numerical simulation of stochastic differential equations. *SIAM Review*, 43(3):525–546, 2001.

[11] Peter E. Kloeden and Eckhard Platen. *Numerical solution of stochastic differential equations*, volume 23. Springer-Verlag Berlin Heidelberg, Berlin, Heidelberg, 1992.

[12] Darren J Wilkinson. *Stochastic modelling for systems biology.* CRC press, 2011.

[13] Josselin Milloz, Fabien Duveau, Isabelle Nuez, and Marie Anne Félix. Intraspecific evolution of the intercellular signaling network underlying a robust developmental system. *Genes Dev.*, 22(21):3064–3075, 2008.

[14] Tina Toni, David Welch, Natalja Strelkowa, Andreas Ipsen, and Michael PH Stumpf. Approximate bayesian computation scheme for parameter inference and model selection in dynamical systems. *J. R. Soc. Interface*, 6:187–202, 2009.

[15] S A Sisson, Y Fan, and Mark M Tanaka. Sequential Monte Carlo without likelihoods. *Proceedings of the National Academy of Sciences*, 104(6):1760–5, feb 2007.

[16] Mark A Beaumont, Jean-Marie Cornuet, Jean-Michel Marin, and Christian P Robert. Adaptive approximate Bayesian computation. *Biometrika*, 96(4):983–990, 2009.

[17] S A Sisson, Y Fan, and Mark M Tanaka. Correction for Sisson et al., Sequential Monte Carlo without likelihoods. *Proceedings of the National Academy of Sciences*, 106(39):16889–16889, 2009.

[18] Pierre Del Moral, Arnaud Doucet, Ajay Jasra, Monte Carlo, Sequential Monte, Carlo P Del Moral, A Doucet, and A Jasra. An adaptive sequential Monte Carlo method for approximate Bayesian computation. *Stat Comput*, 22:1009–1020, 2012.

[19] Juliane Liepe, Paul Kirk, Sarah Filippi, Tina Toni, Chris P Barnes, and Michael P H Stumpf. A framework for parameter estimation and model selection from experimental data in systems biology using approximate Bayesian computation. *Nat Protoc*, 9:439–456, 2014.

[20] Daniel Silk, Sarah Filippi, and Michael P H Stumpf. Optimizing threshold - schedules for approximate bayesian computation sequential monte carlo samplers: Applications to molecular systems. *arXiv preprint arXiv:1210.3296*, 2012.

[21] Sarah Filippi, Chris P. Barnes, Julien Cornebise, and Michael P.H. Stumpf. On optimality of kernels for approximate Bayesian computation using sequential Monte Carlo. *Statistical Applications in Genetics and Molecular Biology*, 12(1):87–107, 2013.
