# Supplementary figures and images for: Quantifying cell transitions in C. elegans with data-fitted landscape models
Source: PLoS Comput Biol. 2021 Jun 1;17(6):e1009034. doi: 10.1371/journal.pcbi.1009034 (PMC8195438; doi:10.1371/journal.pcbi.1009034)

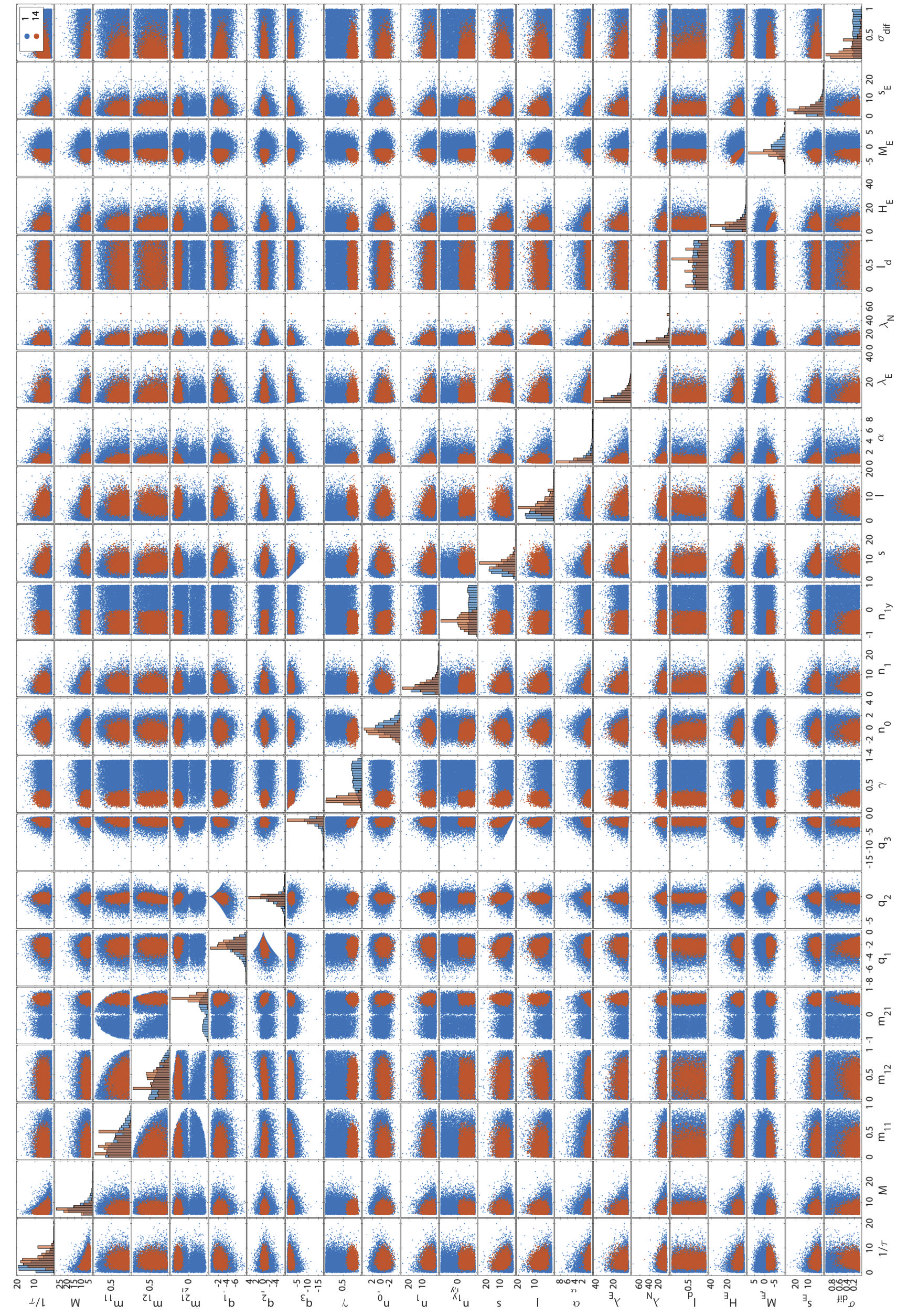

Supplement: S1 Fig — Histograms and two dimensional scatter plots of the N = 2 × 10^4^ particles sampled from the approximated posterior distribution given the training data in Table 1 at the first (blue) and last (red) steps of the algorithm. (TIF) [file pcbi.1009034.s002.tif]

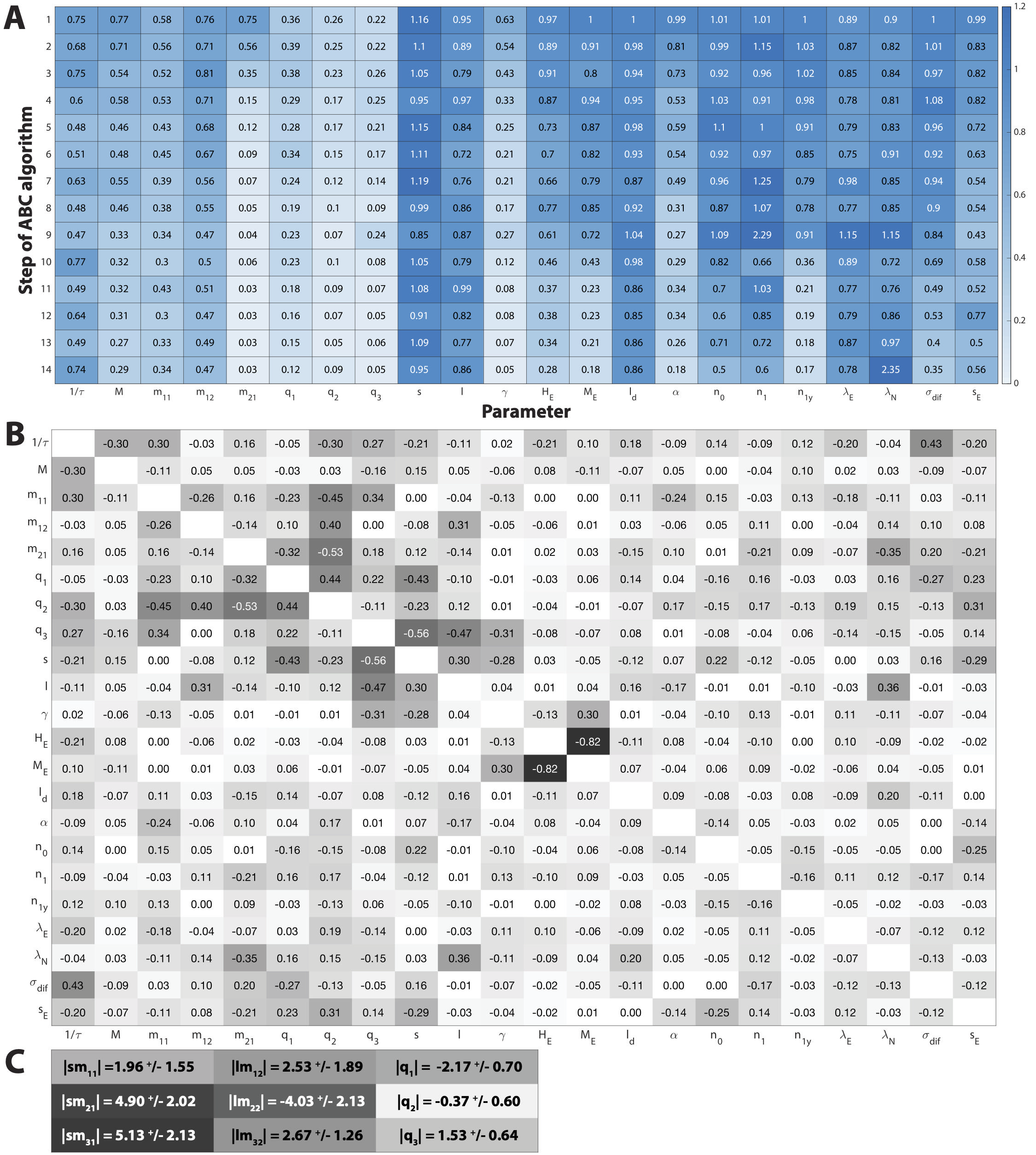

Supplement: S2 Fig — (A) Evolution of the variance of the parameters at each step of the ABC algorithm, normalised by the variance of the prior. (B) Correlation matrix of the parameters at the last step of the algorithm. (C) Approximated posterior distributions of the parameters defining the linear transformation of the signal space into the control space. (TIF) [file pcbi.1009034.s003.tif]

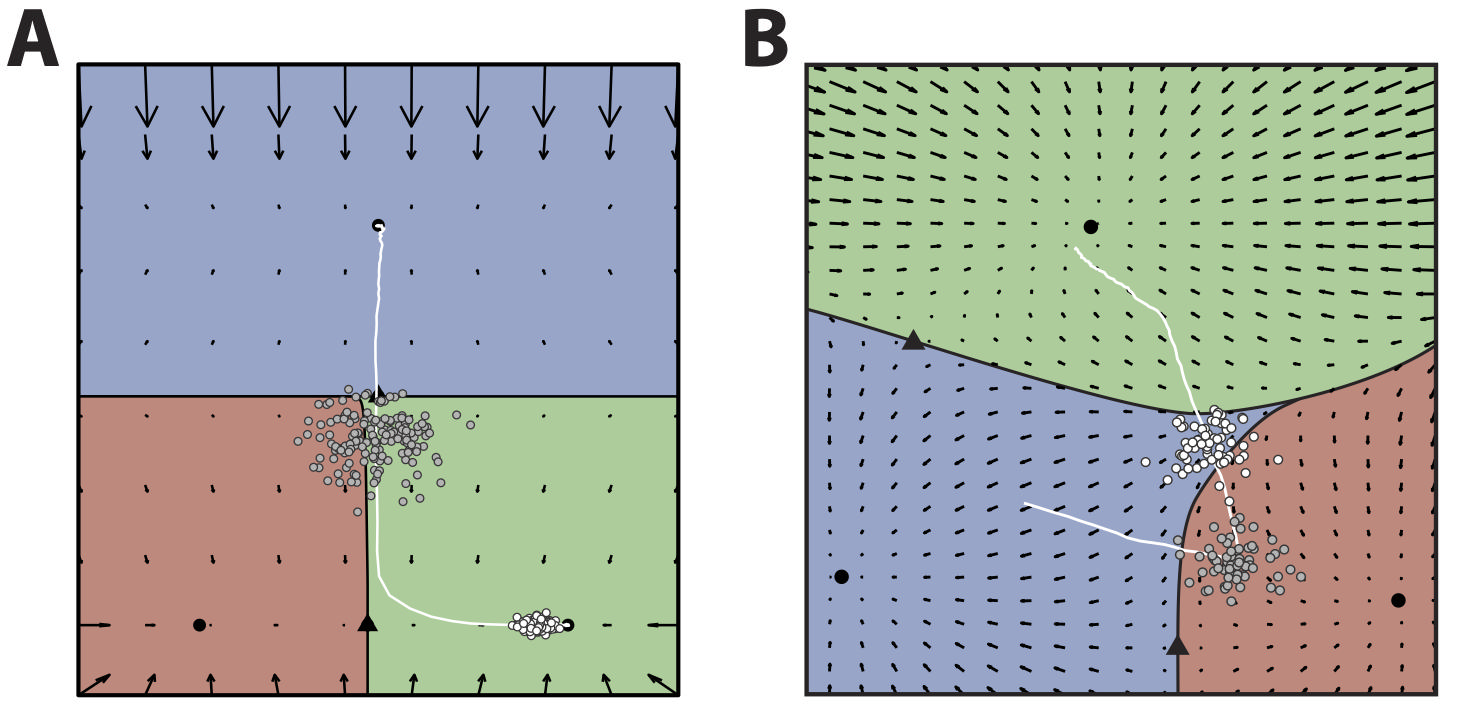

Supplement: S3 Fig — (A) Simulations of the differentiation of isolated VPCs exposed to EGF from time t1 = 0 to t2 = 0.4 followed by Notch from t2 = 0.4 to t3 = 1 following the binary flip with cusp model, plotted on the landscape corresponding to the absence of signaling. The mean trajectory of VPCs is represented as a white line. The distribution of cell states at time t = 0.4 is shown as gray dots. The distribution of cell states at time t = 0.62 is shown as white dots. (B) Simulations of the differentiation of isolated VPCs exposed to EGF from time t1 = 0 to t2 = 0.4 followed by Notch from t2 = 0.4 to t3 = 1 following the model proposed in [3, 5], plotted on the landscape corresponding to the absence of signaling. The mean trajectory of VPCs is represented as a white line. The distribution of cell states at time t = 0.4 is shown as gray dots. The distribution of cell states at time t = 0.62 is shown as white dots. Attractors and saddles are represented by black dots and triangles, respectively. Basins of attraction are colored blue, green or red if they represent tertiary, secondary or primary fates, respectively. (TIF) [file pcbi.1009034.s004.tif]
