# Supplementary material for: Quantifying cell transitions in C. elegans with data-fitted landscape models
Source: PLoS Comput Biol. 2021 Jun 1;17(6):e1009034. doi: 10.1371/journal.pcbi.1009034 (PMC8195438; doi:10.1371/journal.pcbi.1009034)
Supplement: Attachment — Submitted filename: Response_to_Reviewers.pdf [file pcbi.1009034.s019.pdf]

We thank all the reviewers for their careful reading of our manuscript and their positive feedback as well as constructive criticisms and suggestions. We implemented the changes that we detail in this response as well as modified the format of the manuscript and added an Author Summary.

Here is a point-by-point response to the reviewers' comments and concerns.

Reviewer #1: In short, I consider this to be an outstanding paper. The authors combine ideas from catastrophe theory — an area that has been around a fair while, but which has rarely been employed to understand real systems beyond simply pointing out similarities in a rather hand-waving fashion — with (approximate) Bayesian inference employing real data. They employ this to a classical developmental model system, the vulva development in C elegans.

It builds on and significantly improves upon recent work which established more formal analysis of Waddington's epigenetic landscape and developmental systems.
I did like the systematic development of a model that encapsulates the desired cell fate transitions/bifurcations: it was remarkably clear to follow and each step is explained with sufficient detail to be reproducible.

Model construction is well described and the authors then attempt to calibrate the model against data using ABC. The approach taken by the authors is again clearly motivated and explained. One question that I would like to be addressed more clearly is the choice of distance function, Eqn. (10). The second term is well explained, but in comparing the probabilities, other alternative distances, in particular information theoretical distances could have been considered? Such a discussion could go into Appendix 2.

We thank the reviewer for this comment. We would like to point out that we did not have access to the primary data and only had access to the summary statistics presented in Table 1 of the main text. Therefore, distance measures based on information-theoretic quantities such as the KL divergence or mutual information could not be used. We did not run any optimization with an alternative distance and so cannot comment on this in the current manuscript, but we agree with the reviewer that considering information-based measures where primary data is available would be a very interesting future direction.

The way in which the model is calibrated against the data is well explained in Figure 4. But I was intrigued by the results of 4B and would like to learn more. The videos accompanying Figure 4 are superb by the way.
From S 4.1 I was also wondering if the authors could provide some indication as to which parameters can be inferred (or are constrained by the data — I also fear that the text is a bit too small in the current resolution).

We thank the reviewer for this comment. We created a figure showing the change in variance for each parameter at each step of the algorithm (normalized by the variance of the prior), we

also made a plot with the correlations between the parameters (S2 Fig) and we have increased the resolution of S1 Fig. We have also added the following paragraph to the main text (lines 414-431), as well as a subsection in Appendix 2 that contains a table with the priors and posteriors of the parameters:
Analysis of the evolution of the approximate posterior distributions of the parameters at each step of the ABC algorithm shows that most of the parameters are very well constrained by the data (\nameref{figsupp:sf1}, \nameref{figsupp:sf2}), with the exception of the scaling parameters s and l that correlated with the parameter q_3 of the linear transformation that determines the position of the fold line. Also, the parameter l_d, which controls a direct coupling between EGF and Notch pathways, was not crucial to reproduce the data, and this is especially important because, as we will show in the following section, the model is able to reproduce epistatic effects without this coupling. Finally, the exponential decays λ_{E} and λ_{N} were only constrained such there was sufficient time to allow the system to return to zero-signal condition (\nameref{first:app}). Interestingly, the noise magnitude represented by the parameter σ_{dif} was strongly constrained, suggesting that the data strongly constrained noise-driven fluctuations. Moreover, the fitting did not show strong parameter correlations with the exception of parameters H_E and M_E, defining the sigmoidal increase of EGF signal in time. Also, it is important to note that the parameters γ and $\mathbf{n}_1$ that control the balance between the morphogen and sequential models were strongly constrained. In particular, $\gamma = 0.22\pm 0.06<1$ and $\mathbf{n}_1$ pointed towards first fate, so we can infer from the data that both morphogen and sequential features were necessary.

In the validation section the authors show that their model can capture epistatic effects. This is an additional appeal of the approach developed by the authors. Figure 6A shows this, but I found the discussion somewhat terse.

We thank the reviewer for this comment. We have expanded the discussion of the validation section for clarity.

A central statement in the discussion is "Moreover, we can expect that the dynamics of any GRN will be largely described by the normal forms that Catastrophe Theory and Dynamical Systems Theory provide." I think I would on balance agree with this fundamentally; but I wish the authors would try to make this point in more detail.

We thank the reviewer for the comment. We have modified the text to include references supporting this statement.

I would also suggest that the authors make a more obvious statement that the methodology is available at a dedicated github site (it is only mentioned in the appendices and in the pdf

not close to any mention of "software" or "methodology". Finally, some indication as to the run-time would be helpful.

We thank the author for the suggestion. We modified the manuscript and added a sentence indicating that the methodology and software are available in the corresponding GitHub repository (lines 382-383).
We also added the following paragraph to the Appendix and to the GitHub repository:
It took a mean of 3.5 seconds to simulate all the mutants in the Training Data set given one particle that satisfied all the constraints and priors. The time to run the ABC algorithm depended on the step of the algorithm and number of particles to be found. For N=20,000 particles, the time increased as the algorithm is challenged with smaller thresholds, ranging from a mean of 3.5 seconds/particle on the first step of the algorithm to a mean of 56.15 seconds/particle on the last step of the algorithm. This means, to compute 20,000 particles with 4 cores or parallel workers it ranged from 4.8 hours on the first step to 78 hours on the last one.

##Minor Comments:
- Line 214 & 223 and throughout: be clearer with your notation x, y, y_1, y_i. What is the range of index i?

We thank the reviewer for this comment. We have modified the text to be more specific (lines 213-214 and 223-225).

- Line 593: "article to be submitted" is not a suitable reference.

We thank the reviewer for this comment, we have updated the reference to:
Saez, M., Blassberg, R., Camacho-Aguilar, E., Siggia, E. D., Rand, D. A., & Briscoe, J. (2021). A quantitative landscape of cell fate transitions identifies principles of cellular decision-making. *bioRxiv*.

Reviewer #2: In this manuscript, Camacho-Aguilar et al present an analytical formulation for the Waddington landscape to model cell-fate commitment in C.elegans vulval development. To set up such model, they use elements of Catastrophe Theory to combine cusp and fold potentials and create what they call a "binary flip with cusp" landscape. Next, they use approximate Bayesian computation methods to test the universality of their model by fitting it to previously published datasets in which vulval development is investigated under different conditions.

This paper is very exciting to read. The authors make a real effort to present the model system (vulval development), and introduce all their theoretical toolkit (Catastrophe Theory and approximate Bayesian computation). Any reader will benefit from such a pedagogical approach.

I have two major comments that I would like the authors to clarify:

1) **In their Introduction, the authors introduce how the Morphogen and the Sequential models are typically used to explain vulval development. In the results, they state that "*our results suggest that aspects from both the morphogen and the sequential model are important for the correct patterning, which combine into a two-step decision logic determined by the fitted fate map and controlled by the dynamics of the EGF and Notch signals received by the cells*". In my opinion, the binary flip with cusp landscape proposed by the authors already conceptually combines elements from both the Morphogen and the Sequential models, therefore it is not surprising that their results satisfy such framework. Hence, I don't think that the results suggest anything, rather than the model reconciles the two points of view. Could the authors comment on this?**

We thank the reviewer for this comment. Although it is true that the model combines elements from both morphogen and sequential model, there is sufficient flexibility in the parameters to allow the fitting algorithm to choose whether the morphogen model is used or not, as well as the sequential model. If \gamma was equal to 1, there would be no morphogen effect in the system. In the same way, the sequential model is tuned through the parameters of the sigmoidal function in equation 6. In both cases, the data constrained the values of the parameters in a way that both the morphogen model and sequential model need to be combined for the model to fit the data. In particular, \gamma = 0.22 +- 0.06, and n_{1,y} points towards first fate. This is why we state that the model suggests both morphogen and sequential models are involved. We addressed this in the main text (lines 427-431).

2) **The authors based their theoretical approach on previous work by Corson and Siggia. In several parts of the manuscript it is mentioned that the landscape and approach proposed in this work are simpler. However, the two models equally fit the data and, according to the authors, make the same predictions. Can the authors think of an experiment or a prediction that is different between the two theoretical descriptions? Otherwise, other than the Occam's razor, what is the benefit of this new model? Why**

do the authors think that the Waddington landscape should not have repellent states? Being able to address this would provide an impactful interpretation of cell-fate commitment in vulval development.

We thank the reviewer for this comment. We have now added an example that could differentiate between the two theoretical descriptions at the end of the Model's predictions section (copied below), and a supplemental figure (S3 Fig.). The main difference between the two models is the flexibility of the state corresponding to the tertiary fate. In our model, once a cell leaves that state, it is not able to go back. However, as we show, this is possible in the model proposed by Corson and Siggia. We would like to clarify that we don't think the Waddington landscape should not have repellers, but repelling states add a complexity level that is not necessary to model this particular biological process.

Finally, we explored whether there was a prediction that could highlight the differences between the model presented here and that proposed in \cite{siggia,Corson}. An important difference between the two models is the fact that the binary flip with cusp model is not symmetric, in that once cells have sufficiently left the basin of attraction of the tertiary fate they cannot go back for any value of the external signals. For example, if isolated cells (with no autocrine or paracrine Notch signaling) are exposed to a short EGF pulse of the same WT strength (from time $t_1=0$ to $t_2=0.4$), our model predicts that most of them would leave the basin of attraction of tertiary fate and commit to either the primary or secondary fate (\nameref{figsupp:sf3}A). Moreover, due to the directions of the flow lines, there is no signaling profile that would be able to return cells back to tertiary fate. For example, if this short EGF pulse is followed by a very short Notch signaling pulse ($t_2=0.4$ to $t_3=0.62$), cells remain outside the basin of attraction of the tertiary fate and would differentiate into the secondary fate (\nameref{figsupp:sf3}A). However, this is not the case in the model proposed by \cite{siggia,Corson}. As in our model, isolated cells exposed to the same short EGF pulse leave the tertiary fate and commit to primary fate, resulting in $~90\%$ of primary-fated cells (\nameref{figsupp:sf3}B). Strikingly, if this is followed by the short Notch signaling pulse, the cells return to the basin of attraction corresponding to the tertiary fate, resulting in $~71\%$ now differentiating to the tertiary fate and only $~19\%$ differentiating to the primary fate (\nameref{figsupp:sf3}B). Performing this or a similar experiment would be able to differentiate between the two models.

Additionally, I have some minor comments:

3) I assume that the white triangles in Fig. 1B indicate saddle-points. Could the authors clarify this and add this information in the caption?

We thank the reviewer for noticing this, we have now added this information in the caption.

4) Why is the landscape depicted in C2 not a particular case of C3?

We thank the reviewer for this comment. C2 could be a qualitatively a particular case of C3, however, the way we construct C2 gives more freedom as to where in the parameter space the bifurcations happen.

5) **I find the format and the caption in Table 1 confusing and was only able to understand the numbers provided after reading the manuscript twice. The format of the provided information in the upper side is different from the bottom side. The authors mention in the text that the table displays probability measurements, but the numbers span between 0 and 100 (probabilities go from 0 to 1 by definition). Also, the abbreviations VD and TD are explained in the text but absent in the caption.**

We thank the reviewer for this comment. We have changed the first part of the table to match the format of the lower part of the table. Probabilities were converted into percentages for clarity, but we have now added a note in the caption to clarify it. We have also added a note in the caption to clarify what TD and VD mean.

6) **The authors describe the cell state as a 2-dimensional vector, (x(t),y(t)). When introducing the fold and cusp potentials from Catastrophe Theory, they use y_1 and y_i, but they do not define what they are and how they correspond to y_1 and y_i (it is left to the reader to relate those to x and y). Could the authors simplify this? Additionally, why is it required to introduce the derivative of y_i in the model? What is lambda_i? Despite not being essential, I am convinced that this would increase the readability of the manuscript.**

We thank the reviewer for this comment. We introduce the derivative of y_i in order for the equations of the cusp and the fold to match the ones of the complete dynamical system that we define. We have modified the text to more specifically define the subindices i and parameters \lambda_i (lines 212-214 and 223-225).

7) **I think there is a mistake in the equation in Fig. 2A.**

We thank the reviewer for this comment. It is true, it should be “+cy”. We have now corrected this.

8) **In the section “Dependence upon the morphogens”, the authors introduce a set of 4 facts. I think it should be stated that all cells start the process from the same basin of attraction.**

We thank the reviewer for this comment. We have now added this fact earlier in the text on lines 261-262.

9) In this paper, the authors fit their model to experimental data. Priors of all the parameters are provided in Appendix 2 (Table 1), where some discussion about the values obtained is also provided. I would encourage the authors to discuss some of the values and posteriors obtained by the fit to the main text. Is, for example, the parameter c more related to theta_E?

We thank the reviewer for this comment. We have now created a figure showing the change in variance for each parameter at each step of the algorithm (normalized by the variance of the prior), we also made a plot with the correlations between the parameters and a heatmap showing the final linear transformation which, as the reviewer suggests, shows that c is more related to theta_E (S2 Fig). We have also added the following paragraph to the main text (lines 414-431), as well as a subsection in S1 Appendix that contains a table with the priors and posteriors of the parameters:

Analysis of the evolution of the approximate posterior distributions of the parameters at each step of the ABC algorithm shows that most of the parameters are very well constrained by the data (\nameref{figsupp:sf1}, \nameref{figsupp:sf2}), with the exception of the scaling parameters s and I that correlated with the parameter q_3 of the linear transformation that determines the position of the fold line. Also, the parameter I_d, which controls a direct coupling between EGF and Notch pathways, was not crucial to reproduce the data, and this is especially important because, as we will show in the following section, the model is able to reproduce epistatic effects without this coupling. Finally, the exponential decays λ_{E} and λ_{N} were only constrained such there was sufficient time to allow the system to return to zero-signal condition (\nameref{first:app}). Interestingly, the noise magnitude represented by the parameter σ_{dif} was strongly constrained, suggesting that the data strongly constrained noise-driven fluctuations. Moreover, the fitting did not show strong parameter correlations with the exception of parameters H_E and M_E, defining the sigmoidal increase of EGF signal in time. Also, it is important to note that the parameters γ and $\mathbf{n}_1$ that control the balance between the morphogen and sequential models were strongly constrained. In particular, $\gamma = 0.22\pm 0.06<1$ and $\mathbf{n}_1$ pointed towards first fate, so we can infer from the data that both morphogen and sequential features were necessary.

10) In the videos there is a fluctuating gray dot. What is that? Could the authors provide a more complete caption?

We thank the reviewer for this comment. We have now clarified this in the captions of the videos.

Reviewer #3: This is a brilliant paper. It should be published without further changes.

The study of cell fate and differentiation is an ongoing challenge in cell biology. For mathematical modellers it is natural to think of cell states as attractors of a dynamical system, and hence dynamical systems models have been prevalent. In systems biology, models of gene regulatory network that participate in cell fate decisions are widely used. However, systems biology models tend to suffer from overwhelmingly high dimensionality. This high dimensionality seems inevitable, since genomes contain thousands of genes and transcription factors. In this groundbreaking paper, Camacho-Aguilar et al. propose a new method to construct comparatively low-dimensional dynamical systems that can be quantitatively fitted to experimental data. They very convincingly illustrate the utility of the method by applying it to cell state transitions during C. elegans vulval development. Their model can fit a range of results on the rates of cell state transitions under varying experimental conditions, and correctly predicts further results without fitting. The authors further illustrate the utility of their method by predicting outcomes of a few potential future experiments.

I believe the paper makes a big conceptual advance that will be very important to the quantitative study of cell state transitions in many different systems. It really moves the field forward. In addition, it provides new insights C. elegans development. Hence, I strongly recommend the paper for publication.

The paper is very thorough and well written. I particularly like that it introduces relevant concepts on catastrophe theory from scratch, and that simulation codes are available for download. The only comment that I have relates to previous attempts at constructing Waddington-like landscapes. The authors cite some of these. However, it seems they have missed some of the related work by Yogi Jaeger and colleagues, such as (I am not one of the authors): https://bmcsystbiol.biomedcentral.com/articles/10.1186/1752-0509-8-43

We thank the reviewer for this suggestion. We are aware of this work and believe is quite important, as well as other related work by Sui Huang, Yogi Jaeger, Nik Monk, and Michael P.H. Stump. However, in the introduction, we only mention work on landscapes that do not refer to the underlying gene regulatory network. We have added references to this work together with other relevant work to the discussion section (lines 590-596).

I would like to apologise to the authors for the delay in submitting this review.
