# Supplementary material for: Quantifying cell transitions in C. elegans with data-fitted landscape models
Source: PLoS Comput Biol. 2021 Jun 1;17(6):e1009034. doi: 10.1371/journal.pcbi.1009034 (PMC8195438; doi:10.1371/journal.pcbi.1009034)
Supplement: Attachment — Submitted filename: Referee_round1.docx [file pcbi.1009034.s018.docx]

In this manuscript, Camacho-Aguilar et al present an analytical formulation for the Waddington landscape to model cell-fate commitment in C.elegans vulval development. To set up such model, they use elements of Catastrophe Theory to combine cusp and fold potentials and create what they call a "binary flip with cusp" landscape. Next, they use approximate Bayesian computation methods to test the universality of their model by fitting it to previously published datasets in which vulval development is investigated under different conditions.

This paper is very exciting to read. The authors make a real effort to present the model system (vulval development), and introduce all their theoretical toolkit (Catastrophe Theory and approximate Bayesian computation). Any reader will benefit from such a pedagogical approach.

I have two major comments that I would like the authors to clarify:

1) In their Introduction, the authors introduce how the Morphogen and the Sequential models are typically used to explain vulval development. In the results, they state that "*our results suggest that aspects from both the morphogen and the sequential model are important for the correct patterning, which combine into a two-step decision logic determined by the fitted fate map and controlled by the dynamics of the EGF and Notch signals received by the cells*". In my opinion, the binary flip with cusp landscape proposed by the authors already conceptually combines elements from both the Morphogen and the Sequential models, therefore it is not surprising that their results satisfy such framework. Hence, I don’t think that the results suggest anything, rather than the model reconciles the two points of view. Could the authors comment on this?
2) The authors based their theoretical approach on previous work by Corson and Siggia. In several parts of the manuscript it is mentioned that the landscape and approach proposed in this work are simpler. However, the two models equally fit the data and, according to the authors, make the same predictions. Can the authors think of an experiment or a prediction that is different between the two theoretical descriptions? Otherwise, other than the Occam’s razor, what is the benefit of this new model? Why do the authors think that the Waddington landscape should not have repellent states? Being able to address this would provide an impactful interpretation of cell-fate commitment in vulval development.

Additionally, I have some minor comments:

3) I assume that the white triangles in Fig. 1B indicate saddle-points. Could the authors clarify this and add this information in the caption?
4) Why is the landscape depicted in C2 not a particular case of C3?
5) I find the format and the caption in Table 1 confusing and was only able to understand the numbers provided after reading the manuscript twice. The format of the provided information in the upper side is different from the bottom side. The authors mention in the text that the table displays probability measurements, but the numbers span between 0 and 100 (probabilities go from 0 to 1 by definition). Also, the abbreviations VD and TD are explained in the text but absent in the caption.
6) The authors describe the cell state as a 2-dimensional vector, (x(t),y(t)). When introducing the fold and cusp potentials from Catastrophe Theory, they use y_1 and y_i, but they do not define what they are and how they correspond to y_1 and y_i (it is left to the reader to relate those to x and y). Could the authors simplify this? Additionally, why is it required to introduce the derivative of y_i in the model? What is lambda_i? Despite not being essential, I am convinced that this would increase the readability of the manuscript.
7) I think there is a mistake in the equation in Fig. 2A.
8) In the section “Dependence upon the morphogens”, the authors introduce a set of 4 facts. I think it should be stated that all cells start the process from the same basin of attraction.
9) In this paper, the authors fit their model to experimental data. Priors of all the parameters are provided in Appendix 2 (Table 1), where some discussion about the values obtained is also provided. I would encourage the authors to discuss some of the values and posteriors obtained by the fit to the main text. Is, for example, the parameter c more related to theta_E?
10) In the videos there is a fluctuating gray dot. What is that? Could the authors provide a more complete caption?
